# Early Tumor Shrinkage and Depth of Response as Predictive Markers of Treatment Response and Prognosis in Solid Tumors

**DOI:** 10.1002/cam4.71251

**Published:** 2025-09-17

**Authors:** Peng Cao, Xiaojuan Yang

**Affiliations:** ^1^ Department of Colorectal Cancer Center West China Hospital, Sichuan University Chengdu China; ^2^ Liver Digital Transformation Research Laboratory, State Key Laboratory of Biotherapy and Cancer Center, West China Hospital Sichuan University and Collaborative Innovation Center of Biotherapy Chengdu Sichuan People's Republic of China

**Keywords:** DpR, ETS, OS, PFS, solid tumor

## Abstract

**Background:**

Evaluation of tumor efficacy is central to cancer care. Progression‐free survival (PFS) is widely used as an early surrogate for treatment effectiveness, but more timely and reliable biomarkers are needed. Early tumor shrinkage (ETS) and depth of response (DpR) have emerged as promising predictors: ETS reflects early treatment sensitivity at first radiologic assessment, whereas DpR quantifies the maximum tumor reduction and may capture the durability of benefit.

**Methods:**

We conducted a narrative synthesis of clinical studies assessing ETS and/or DpR across solid tumors, focusing on their definitions, measurement under RECIST, and associations with PFS and overall suvival (OS). We also summarized advances in imaging and multidimensional assessment framworks that could improve the accuracy and clinical utility of these indicators, and highlighted sources of heterogeneity and current gaps.

**Results:**

Across multiple retrospective and post‐hoc analyses, ETS provides an early signal that identifies patients more likely to benefit from therapy and can inform treatment adaptation. DpR shows consisten correlations with long‐term outcomes and complements PFS by reflecting the magnitude of tumor control. Both ETS and DpR demonstrate predictive value for PFS and OS; however, variability in cut‐offs (e.g., ETS 20%–30%), timing of assessments, tumor types, and treatment modalities limits comparability. Emerging imaging technologies and composite response frameworks offer opportunities to enhance measurement precision and reproducibility.

**Conclusions:**

ETS and DpR are promising, clinically interpretable markers for monitoring treatment effcacy and prognosis. Standardized definitons, prospective validation, and integration with molecular and imaging biomarkers (e.g., ctDNA, radiomics, and machine—learning—enhanced imaging) are needed to refine their application and solidify their role in routine cancer therapy monitoring.

## 1 Introduction

In systemic therapy, treatment efficacy is typically assessed using indicators such as objective response rate (ORR), progression‐free survival (PFS), and overall survival (OS). Among these, PFS serves as a valuable surrogate marker for OS, offering the advantage of reflecting treatment effects at an earlier stage, often before changes in OS become apparent [1, 2]. However, with the continuous advancement of cancer therapies, both PFS and OS have significantly improved. As a result, traditional indicators are becoming insufficient for early evaluation of treatment efficacy. Therefore, there is an urgent need to identify novel biomarkers that are not only easier to obtain and simpler to assess, but also capable of providing earlier insights into treatment response and patient prognosis.

In clinical trials, tumor response is typically assessed using the Response Evaluation Criteria in Solid Tumors (RECIST). These criteria offer a standardized framework for evaluating treatment outcomes, enabling clinicians, researchers, and drug developers to consistently describe, assess, and compare the effects of cancer therapies. RECIST evaluates treatment response by measuring changes in the maximum diameter of target lesions (the sum of the longest diameters, SLD) and classifies tumors into four categories: complete response (CR), partial response (PR), disease progression (PD), and stable disease (SD). Specifically, CR is defined as the complete disappearance of all lesions, PR as a reduction of at least 30% in lesion size, PD as an increase of at least 20% in lesion size, and SD as a reduction of less than 30% or an increase of less than 20%. The disease control rate (DCR) is calculated as the sum of patients achieving CR, PR, and SD [3].

Despite the significant role of the RECIST criteria in tumor efficacy assessment, it has notable limitations. In particular, for patients classified as having SD, survival times can vary substantially. Some studies suggest that, in patients with advanced nonsmall cell lung cancer (NSCLC), the DCR at week 8 may be a better predictor of long‐term survival than CR or PR [4]. Therefore, more accurate methods for assessing the prognosis of these patients—guiding clinical treatment decisions—remain an urgent challenge. Additionally, the RECIST criteria evaluate efficacy at fixed time points and are unable to capture dynamic tumor changes in real time, leading to delayed assessment of treatment responses. Furthermore, studies have indicated that the correlation between RECIST and long‐term survival benefits is limited [5]. As a result, emerging metrics such as early tumor shrinkage (ETS) and depth of tumor response (DpR) have become increasingly important tools for assessing treatment efficacy. These metrics offer a more comprehensive and dynamic reflection of both short‐term and long‐term treatment effects, providing strong predictive value for patient survival outcomes.

As an early treatment response indicator, ETS enables the rapid assessment of a patient's sensitivity to treatment in the early stages, providing a foundation for the dynamic adjustment of treatment strategies [6, 7]. On the other hand, DpR focuses on quantifying treatment efficacy by measuring the maximum extent of tumor shrinkage. It has been considered an important endpoint, as it may serve as a surrogate for evaluating PFS and/or OS in current clinical trials [8]. Existing studies have shown that both ETS and DpR are closely associated with long‐term survival outcomes in cancer patients. By combining short‐term and long‐term analyses, this approach provides a more comprehensive evaluation of treatment efficacy. In pan‐cancer analyses, ETS and DpR not only enhance tumor efficacy assessment but also reveal variations across different cancer types, highlighting the relationship between various therapeutic agents and tumor growth dynamics. As continuous variables, the optimal cutoff values for ETS and DpR remain under investigation. This review will focus on the application of ETS and DpR in pan‐cancer contexts and explore the optimization of their cutoff values. Furthermore, with advances in imaging technologies, parameters, and biomarkers, the predictive power of ETS and DpR will be further strengthened, supporting the development of personalized treatments and optimizing cross‐cancer efficacy.

## 2 ETS

**FIGURE 1:**
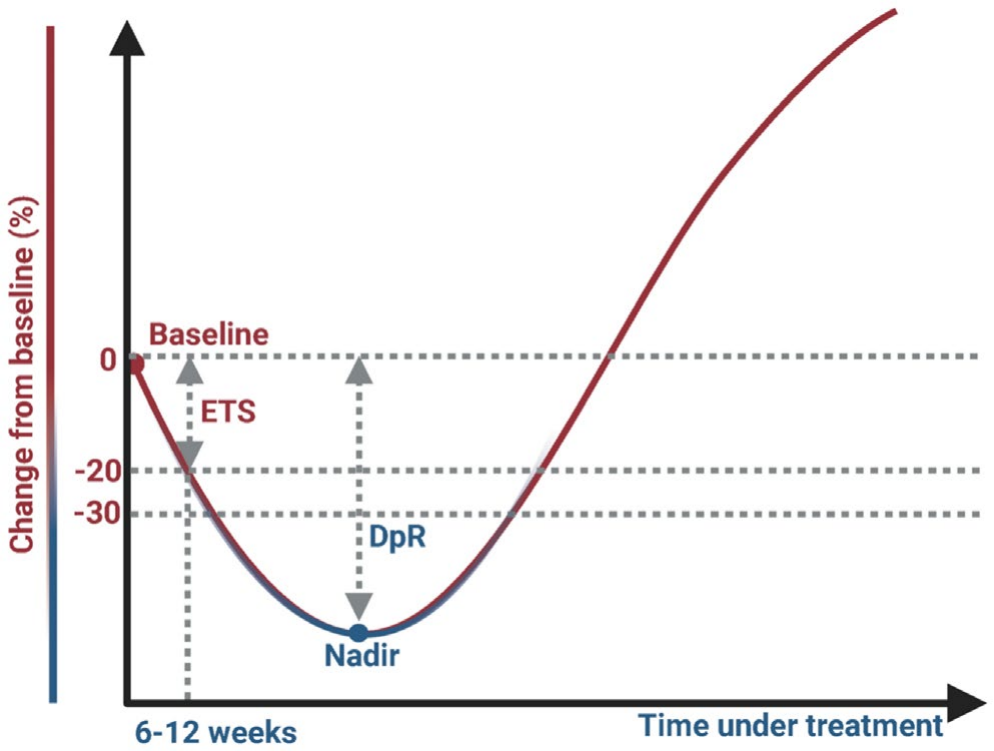
Definition of early tumor shrinkage (ETS) and depth of response (DpR).

ETS serves as an early indicator for assessing treatment sensitivity, typically referring to the degree of tumor volume reduction from baseline during the initial stages of treatment [9]. The evaluation is typically performed 6 to 12 weeks after treatment initiation [9, 10, 11]. (Figure 1) ETS is a continuous variable, but in most studies, it is often categorized into binary variables based on a cutoff value to evaluate its predictive effect on PFS and OS in responders versus nonresponders. ETS assessment is typically combined with imaging tools (such as CT or MRI) and follows the RECIST criteria, which assess the percentage reduction in the sum of the longest diameters (SLD) of target lesions from baseline. According to previous retrospective studies, common ETS thresholds are reductions in SLD of 10% to 20% [9, 12], Although specific percentages may vary depending on the treatment regimen and cancer type, these thresholds provide a useful framework for evaluating ETS. However, a unified standard for ETS thresholds has yet to be established. ETS is regarded as an early indicator of treatment sensitivity, capable of quickly distinguishing between patients who respond to treatment and those who do not. Studies have shown that achieving ETS is significantly associated with longer PFS and OS [9, 11, 13], although heterogeneity exists across different tumor types [14]. Compared to the traditional RECIST criteria, ETS places greater emphasis on dynamic short‐term changes, offering clear efficacy signals early in treatment and providing valuable insights for treatment optimization and patient management.

## 3 DpR

DpR refers to the maximum extent of tumor regression observed during treatment (Figure 1). The assessment of DpR is based on CT or MRI scans, typically evaluated using the RECIST criteria, and measures the greatest reduction in the SLD of the tumor compared to baseline. DpR is a continuous variable used to quantify a patient's treatment response, reflecting the overall extent of tumor regression. Current research indicates that a greater DpR is generally associated with longer PFS and OS [10, 15]. Compared to ETS, DpR places greater emphasis on predicting long‐term treatment efficacy. By quantifying the maximum extent of tumor regression, DpR provides additional long‐term survival information not captured by the traditional RECIST criteria. It offers a comprehensive assessment of the depth of tumor control induced by treatment, providing valuable insights for precision therapy and patient management.

## 4 Methods

A systematic literature search was conducted to identify relevant studies evaluating the prognostic and predictive value of ETS and DpR across solid tumors. The search was performed in PubMed, Embase, Web of Science, and the Cochrane Library, covering publications from database inception to December 2024. Search terms included a combination of controlled vocabulary and free‐text keywords: “early tumor shrinkage” OR “ETS” OR “depth of response” OR “DpR” AND “progression‐free survival” OR “PFS” OR “overall survival” OR “OS” AND “solid tumor” OR specific tumor types (e.g., “lung cancer,” “colorectal cancer,” “gastric cancer,” “renal cell carcinoma,” “pancreatic cancer”). Reference lists of relevant articles were also manually screened to identify additional eligible studies.

Inclusion criteria were: (1) original clinical trials (prospective or retrospective) or meta‐analyses that evaluated ETS and/or DpR in solid tumors; (2) studies reporting at least one survival outcome (PFS and/or OS) in relation to ETS or DpR; and (3) publications in English. Exclusion criteria were: (1) case reports, reviews, editorials, conference abstracts, or preclinical studies; (2) studies lacking explicit definitions or cut‐off values for ETS/DpR; and (3) insufficient survival data for analysis.

Two investigators independently screened titles and abstracts, and any discrepancies were resolved by consensus. Data were extracted on study design, patient population, tumor type, treatment regimen, ETS/DpR definition, cutoff values, and associations with survival outcomes.

## 5 Application of ETS and DpR in Pan‐Cancer

### 5.1 Lung Cancer

Immune checkpoint inhibitors are commonly used in the treatment of lung cancer. Since immune therapy responses differ from those of conventional chemotherapy and tyrosine kinase inhibitors (TKIs), such as pseudoprogression, exploring more reliable and early indicators of treatment efficacy is crucial for guiding therapy. In a retrospective study, researchers summarized four randomized clinical trials—OAK, POPLAR, BIRCH, and FIR—to explore the relationship between ETS and PFS and OS. OAK and POPLAR focused on advanced nonsmall cell lung cancer (NSCLC) patients who failed platinum‐based therapy and were randomized to receive either 1200 mg of atezolizumab or 75 mg/m^2^ docetaxel every 3 weeks. BIRCH and FIR focused on advanced PD‐L1 positive NSCLC patients who received 1200 mg of atezolizumab every 3 weeks. By analyzing data from the OAK and POPLAR trials, the study identified the cutoff for ETS: a 10% reduction in SLD of target lesions by the sixth week. Both univariate and multivariate analyses suggested that ETS is an independent predictor of PFS and OS, with better prognosis linked to ETS vs. non‐ETS (OS: HR 0.32 95% confidence interval (CI) (0.23–0.45), *p* < 0.001, PFS: HR 0.31 95% CI (0.24–0.40), *p* < 0.001). Moreover, the immune therapy group showed greater benefit from ETS on PFS and OS compared to the docetaxel group. This finding was further tested in the BIRCH and FIR studies, where similar conclusions were reached. This suggests that ETS could serve as a marker of benefit from immune therapy [12]. In another study involving nivolumab treatment for NSCLC patients, researchers defined ETS as the percentage of tumor reduction observed between 8 to 12 weeks after starting nivolumab treatment, and evaluated its correlation with survival outcomes. They found that when ETS was > 10%, it correlated with improved PFS and OS, providing a strong predictive value for patient prognosis. Similar conclusions were obtained in atezolizumab treatment [16]. Notably, patients who were classified as SD according to RECIST criteria could still be distinguished based on ETS, identifying those with better prognosis. This suggests that ETS might be more effective at identifying patients who could benefit from immune therapy, even among those classified as SD by RECIST.

DpR, as a predictor of PFS and OS, has also been validated in studies involving pembrolizumab combined with chemotherapy. A retrospective study of 349 NSCLC patients with PD‐L1 tumor proportion score (TPS) ≥ 50% who received immune checkpoint inhibitor‐based first‐line therapy found that higher DpR was associated with longer PFS and OS. When divided into quartiles (Q1: 0%–25%, Q2: 25%–50%, Q3: 50%–75%, Q4: 75%–100%), the study showed that in the high PD‐L1 expression group (> 50%), patients in the Q4 quartile showed better PFS than those in the chemotherapy combination group [17]. This suggests that combining DpR with certain immune markers could improve the ability to differentiate subgroups of patients who are likely to benefit from immune therapy. Tyrosine kinase inhibitors (TKIs) are commonly used in patients with driver gene mutations. Researchers have explored the relationship between DpR and PFS/OS in NSCLC patients treated with ALK inhibitors (ALKi) or PD‐1 antibodies (Ab). This study reviewed data from two randomized controlled trials on ALKi treatment and two on PD‐1 Ab treatment. Patients were divided into quartiles based on DpR (1%–25%, 26%–50%, 51%–75%, 76%–100%). In the ALKi treatment group, Cox analysis showed that all quartiles were significantly associated with PFS, while only the 51% to 75% group and 76% to 100% group showed a statistically significant correlation with OS. As DpR increased, both PFS and OS improved. In the PD‐1 Ab treatment group, all DpR quartiles were significantly associated with PFS and OS, with DpR > 50% showing significantly better prognosis than ≤ 50% [15]. The differences in predictive performance between treatment groups may be due to the differing mechanisms of tumor shrinkage induced by various drugs. The study suggests that a simple quartile approach might not effectively differentiate between patients, and further research is needed to explore the optimal cutoff for DpR.

In studies primarily focused on TKIs therapy, one investigation explored the relationship between DpR and survival outcomes in epidermal growth factor receptor (EGFR)–TKIs treated NSCLC patients. The study analyzed data from multiple randomized clinical trials, including EURTAC, IPASS, ENSURE, LUX‐Lung 3, and LUX‐Lung 6. After 6 and 12 weeks of treatment, tumor size changes from baseline were assessed as depth of response (DpR). The researchers then analyzed the correlation between DpR, PFS, and OS. However, no significant association was found between DpR and either PFS or OS. The study concluded that DpR cannot reliably predict long‐term survival outcomes in EGFR‐TKI treatment. Therefore, in clinical practice, DpR should not be used as a prognostic marker for long‐term outcomes in EGFR‐mutated NSCLC patients [14]. This finding appears to contradict most studies that support the use of DpR as a predictor for PFS and OS. However, in this study, only 71.2% of patients achieved maximum response by the 6‐week evaluation, and 19.4% had not reached the maximum response by 12 weeks, which may explain the conflicting conclusions between this study and the majority of other studies. Additionally, the application of ETS and DpR may need to be tailored to specific populations, as differences in the mechanisms of tumor shrinkage between EGFR‐TKIs and immune therapies could influence the predictive performance of ETS and DpR. In another retrospective study of 265 EGFR‐mutant advanced NSCLC patients, a significant correlation was found between DpR and PFS. Patients were divided into four groups based on their DpR (Q1: 1%–25%, Q2: 26%–50%, Q3: 51%–75%, Q4: 76%–100%). The study found that the greater the DpR, the longer the PFS (Log‐rank *p* < 0.0001). Compared to patients in Q1, those in the Q2, Q3, and Q4 groups had HRs for PFS of 0.58 (95% CI, 0.42–0.80), 0.49 (95% CI, 0.35–0.69), and 0.33 (95% CI, 0.22–0.50), respectively. DpR as a continuous variable was also associated with prolonged PFS (HR 0.20 95% CI (0.13–0.33) *p* < 0.001), suggesting that DpR can serve as an independent predictor for PFS [18]. The study also found that abnormal levels of lactate dehydrogenase (LDH) were associated with worse prognosis, and combining these indicators could improve the prediction of patient outcomes and guide treatment decisions. However, the study did not provide an OS analysis, which would have added further evidence to support these findings.

In chemotherapy‐treated patients, DpR has also been shown to predict survival. A retrospective analysis of the Phase 3 CA031 trial, which involved 959 NSCLC patients receiving carboplatin combined with albumin‐bound paclitaxel or solvent‐based paclitaxel, evaluated the correlation between DpR and OS. Based on tumor shrinkage, patients were divided into quartiles: NTR (0%), Q1 (0%–25%), Q2 (25%–50%), Q3 (50%–75%), and Q4 (75%–100%). The median OS values for the NTR (no tumor reduction), Q1, Q2, Q3, and Q4 groups were 4.8, 10.4, 14.5, 19.3, and 23.5 months, respectively. The study found that DpR was an independent predictor of improved OS, with the HR decreasing from 0.43 to 0.16 as tumor shrinkage increased [19].

In summary, many studies in lung cancer treatment have explored the use of ETS and DpR as surrogate markers for predicting patient survival and treatment efficacy, to guide treatment decisions. Overall, these markers are reasonable and reliable, providing early indications of treatment efficacy and predicting survival. Compared to PFS and OS, ETS and DpR are more easily accessible and measurable. Given the differences in treatment mechanisms and tumor shrinkage patterns across therapies, combining these markers with additional predictive biomarkers could improve the ability to differentiate patient subgroups who are most likely to benefit from treatment Table 1.

**TABLE 1:** ETS and DpR in lung cancer.

Study	Regiment	ETS						DpR				
		ETS Time	Cut off	PFS (ETS vs. non‐ETS)	OS (ETS vs. non‐ETS)	mPFS (month) (ETS vs. non‐ETS)	mOS (month) (ETS vs. non‐ETS)	Cut off	PFS (DpR vs. non‐DpR)	OS (DpR vs. non‐DpR)	mPFS (month) (DpR vs. non‐DpR)	mOS (month) (DpR vs. non‐DpR)
OAK, POPLAR [12]	Atezolizumab/Docetaxel	6 weeks	≥ 10%	HR 0.31 95% CI (0.24–0.40) *p* < 0.001	HR 0.32 95% CI (0.23–0.45) *p* < 0.001							
Daichi Fujimoto [16]	Nivolumab	8–12 weeks	≥ 10%		—	16.6 vs. 5.1						
Kazandjian [15]	ALK inhibitor							Q1 (1%–25%), Q2 (26%–50%), Q3 (51%–75%), Q4 (76%–100%)	Q1:HR 0.19 95% CI (0.09–0.40), Q2:HR 0.11 95% CI (0.06–0.24), Q3:HR 0.05 95% CI (0.03–0.11), Q4:HR 0.03 95% CI (0.02–0.07)	Q1:HR 0.94 95% CI (0.34–2.61), Q2:HR 0.56 95% CI (0.21–1.51), Q3:HR 0.28 95% CI (0.11–0.73), Q4:HR 0.05 95% CI (0.01–0.28)		
	PD‐l Antibody							Q1 (1%–25%), Q2 (26%–50%), Q3 (51%–75%), Q4 (76%–100%)	Q1:HR 0.30, 95% CI (0.22–0.41), Q2:HR 0.22 95% CI (0.15–0.32), Q3:HR 0.09 95% CI (0.06–0.15), Q4:HR 0.07 95% CI (0.03–0.12)	Q1:HR 0.52 95% CI (0.37–0.74), Q2:HR 0.47 95% CI (0.30–0.74), Q3:HR 0.07 95% CI (0.03–0.18), Q4:HR 0.14 95% CI (0.06–0.32)		
Tadaaki Yamada [17]	Pembrolizumab/Pembrolizumab+chemo‐therapy							Q1 (0%–25%), Q2 (25%–50%), Q3 (50%–75%), Q4 (> 75%)	Q1:HR 0.37 95% CI (0.24–0.57), Q2:HR 0.13 95% CI (0.09–0.20), Q3:HR 0.14 95% CI (0.09–0.23), Q4:HR 0.05 95% CI (0.03–0.10)	Q1:HR 0.51 95% CI (0.32–0.81), Q2:HR 0.30 95% CI (0.20–0.45), Q3:HR 0.21 95% CI (0.13–0.35), Q4:HR 0.08 95% CI (0.04–0.15)	< 0: 2.1 Q1: 6.4 Q2: 14.6 Q3: 12.2 Q4: 39.1	< 0: 5.5 Q1: 19.2 Q2: 27.0 Q3: 26.6 Q4: 41.9
Yuan‐Kai Shi [18]	EGFR‐TKI:Gefitinib, Icotinib, Erlotinib							Q1 (1%–25%), Q2 (26%–50%), Q3 (51%–75%), Q4 (76%–100%)	HR 0.62 95% CI (0.54–0.71) *p* < 0.001		Q1: 6.8, Q2: 13.0 Q3: 13.7 Q4: 19.4	
CA031 [19]	Carboplatin + nab‐paclitaxel/Carboplatin + solvent‐based paclitaxel							NTR, Q1 (0%–25%), Q2 (25%–50%), Q3 (50%–75%), Q4 (> 75%)	NTR: reference Q1:HR 0.23 95% CI (0.17–0.30), *p* < 0.0001, Q2:HR 0.18 95% CI (0.13–0.23), *p* < 0.0001, Q3:HR 0.13 95% CI (0.09–0.18), *p* < 0.0001, Q4:HR 0.09 95% CI (0.05–0.15), *p* < 0.0001	NTR: reference Q1:HR 0.43 95% CI (0.34–0.55), *p* < 0.0001, Q2:HR 0.27 95% CI (0.21–0.35), *p* < 0.0001, Q3:HR 0.20 95% CI (0.15–0.28), *p* < 0.0001, Q4:HR 0.16 95% CI (0.10–0.27), *p* < 0.0001	12‐month PFS: NTR: 2.7 (6%) Q1: 5.6 (24%) Q2: 6.9 (24%) Q3: 8.3 (46%) Q4: 10.9 (48%)	NTR: 4.8 Q1: 10.4 Q2: 14.5 Q3: 19.3 Q4: 23.5

Abbreviations: 95% CI, 95% confidence interval; DpR, depth of response; ETS, early tumor shrinkage; HR, Hazard Ratio; mOS, median overall survival; mPFS, median progression free survival; NTR, no tumor reduction; Q, quartile.

### 5.2 Colorectal Cancer

The predictive value of ETS and DpR has been widely examined in metastatic colorectal cancer (mCRC), particularly in the context of chemotherapy combined with targeted agents. Across multiple phase II and III trials, such as TRIBE [9], FIRE‐3 [20], CRYSTAL [21], and OPUS [21], ETS—commonly defined as a ≥ 20% reduction in tumor size within 6 to 8 weeks—was consistently associated with improved PFS and OS. This underscores ETS as a reliable early indicator of treatment sensitivity and long‐term outcome.

DpR, reflecting the maximal degree of tumor shrinkage, has also demonstrated strong prognostic significance. Deeper responses have been associated with superior survival, especially in patients receiving anti‐EGFR therapy (cetuximab or panitumumab) [20, 21, 22, 23, 24, 25]. In FIRE‐3 and related trials, cetuximab‐treated patients not only exhibited higher ETS and DpR rates but also achieved longer OS compared with those treated with bevacizumab, suggesting that ETS and DpR may help distinguish therapeutic benefit between biologic agents in RAS wild‐type disease [20, 21, 23].

However, the utility of these markers is not uniform across all therapeutic settings. While both ETS and DpR have shown prognostic relevance under anti‐VEGF therapy [9, 23, 24, 25, 26], they were less effective in differentiating the efficacy of different chemotherapy backbones. In immunotherapy‐treated MSI‐H/dMMR patients, ETS (≥ 1%) and DpR (≥ 50%) also correlated with improved outcomes [10], but challenges such as pseudoprogression remain unresolved [27]. Furthermore, a meta‐analysis revealed only a weak correlation between DpR and OS at the trial level [28], limiting its utility as a surrogate endpoint.

In summary, ETS and DpR provide clinically meaningful prognostic information in mCRC, with particular value in anti‐EGFR‐based regimens. While promising as early markers of response, their optimal cutoff values remain unsettled, and they cannot yet replace OS in clinical trials. Future research should focus on harmonizing definitions, integrating ETS and DpR with molecular and imaging biomarkers, and clarifying their predictive role across therapeutic modalities Table 2.

**TABLE 2:** ETS and DpR in colorectal cancer.

Study	Regiment	ETS						DpR				
		ETS time	Cut off	PFS (ETS vs. non‐ETS)	OS (ETS vs. non‐ETS)	mPFS (month) (ETS vs. non‐ETS)	mOS (month) (ETS vs. non‐ETS)	Cut off	PFS (DpR vs. non‐DpR)	OS (DpR vs. non‐DpR)	mPFS (month) (DpR vs. non‐DpR)	mOS (month) (DpR vs. non‐DpR)
TRIBE [9]	FOLFOXIRI+Bevacizumab/FOLFIRI+Bevacizumab	8 weeks	≥ 20%	HR 0.70 95% CI (0.53–0.91), *p* = 0.009	HR 0.58 95% CI (0.40–0.82), *p* = 0.002	8.8 vs. 7.2	31.9 vs. 21.9	I quintile, II quintile, III quintile IV quintile, V quintile	HR 0.86 95% CI (0.80–0.92), *p* < 0.001	HR 0.77 95% CI (0.70–0.84), *p* < 0.001	I: 6.0 II: 8.3 III: 6.3 IV: 8.7 V: 13.0	I: 16.7 II: 27.1 III: 29.7 IV: 26.9 V: 39.7
FIRE‐3 [20]	FOLFIRI + cetuximab	8 weeks	≥ 20%	HR 0.59 95% CI (0.41–0.85), *p* = 0.0037	HR 0.52 95% CI (0.34–0.80), *p* = 0.0023	9.7 vs. 5.8	38.3 vs. 20.5					
	FOLFIRI + bevacizumab			HR 0.71 95% CI (0.52–0.97), *p* = 0.03	HR 0.49 95% CI (0.35–0.71), *p* < 0.0001	11.7 vs. 8.3	31.9 vs. 21.2					
CRYSTAL [21]	Chemotherapy + cetuximab	8 weeks	≥ 20%	HR 0.32 95% CI (0.22–0.45), *p* < 0.001	HR 0.53 95% CI (0.40–0.69), *p* < 0.001	14.1 vs. 7.3	30 vs. 18.6					
OPUS [21]				HR 0.22 95% CI (0.11–0.47), *p* < 0.001	HR 0.43 95% CI (0.23–0.78), *p = 0*.006	11.9 vs. 5.7	26 vs. 15.7					
WJOG6210G [23]	FOLFIRI + panitumumab	8 weeks	≥ 20%	HR 0.40 95% CI (0.19–0.79) *p =* 0.009	HR 0.48 95% CI (0.23–0.98) *p =* 0.044	10.5 vs. 5.4	22.3 vs. 14.5					
	FOLFIRI + bevacizumab			HR 0.078 95% CI (0.004–0.37) *p* < 0.001	HR 0.35 95% CI (0.083–0.99) *p* = 0.048	17.1 vs. 5.6	30.9 vs. 13.2					
PLANET‐TTD [24]	Panitumumab − FOLFOX4	8 weeks	≥ 20%	HR 0.2 95% CI (0.1–0.5) *p* = 0.001	HR 0.3 95% CI (0.1–0.8), *p* = 0.021	14 vs. 3	51 vs. 31					
	Panitumumab − FOLFIRI			HR 0.7 95% CI (0.2–2.2) *p* = 0.548	HR 0.3 95% CI (0.1–1.0), *p* = 0.055	15 vs. 8	52 vs. 8					
Valentino [25]	Maintenance with panitumumab+5‐FU/LV	8 weeks	≥ 20%			13.2 vs. 9.5	30.6 vs. 24.7	34%			14.6 vs. 8	38.6 vs. 19.7
	Maintenance with panitumumab					11.1 vs. 7.7	33.1 vs. 18.7				11.4 vs. 6.5	33.1 vs. 18
PanaMa [26]	FU/FA + Panitumumab							57.1%			11.6 vs. 5.7	28.7 vs. 17.8
	FU/FA										7.5/4.3	42.4/21
Filippo Pietrantonio [10]	PD‐1 Ab or PD‐L1 Ab+CTLA‐4 Ab/PD‐1 Ab or PD‐L1 Ab	8/9 weeks	≥ 1%	HR 0.26 95% CI (0.13–0.52) *p* < 0.001	HR 0.35 95% CI (0.15–0.81) *p* = 0.014			50%	HR 0.13 95% CI (0.06–0.31) *p* < 0.001	HR 0.14 95% CI (0.05–0.41) *p* < 0.001		

Abbreviations: 95% CI, 95% confidence interval; Ab, antibody; DpR, depth of response; ETS, early tumor shrinkage; HR, Hazard Ratio; mOS, median overall survival; mPFS, median progression free survival.

### 5.3 Gastric Cancer

In advanced gastric cancer (AGC), ETS and DpR have been evaluated as early markers of therapeutic efficacy. Analyses from trials such as G‐SOX [29] demonstrated that patients achieving ETS (≥ 20% reduction at 6–8 weeks) experienced significantly longer PFS and OS than non‐ETS patients. DpR, analyzed as a continuous measure, also correlated with survival, indicating that the extent of tumor shrinkage provides prognostic value beyond RECIST‐based classifications [29].

Their predictive role appears particularly relevant in HER2‐positive AGC treated with chemotherapy plus trastuzumab. In this setting, both ETS and DpR effectively stratified patients by survival [30, 31], whereas their prognostic power was less pronounced in HER2‐negative patients receiving chemotherapy alone [30]. These findings suggest that ETS and DpR may be most useful in identifying subgroups that benefit from targeted therapy.

Nevertheless, several challenges remain. Most studies adopted cutoff values (e.g., ETS ≥ 20%) derived from colorectal cancer trials, despite biological and clinical differences between the two malignancies. Given the typically more aggressive course of gastric cancer, more suitable thresholds for ETS and DpR are likely required. Moreover, although both metrics provide early prognostic information, their ability to guide regimen selection remains uncertain [29, 30, 31].

In conclusion, ETS and DpR hold promise as markers of early treatment sensitivity in gastric cancer, particularly in the HER2‐positive population. To enhance their clinical applicability, future studies should define tumor‐specific cutoff values and explore integration with molecular and imaging biomarkers for improved treatment stratification Table 3.

**TABLE 3:** ETS and DpR in gastric cancer.

Study	Regiment	ETS						DpR				
		ETS Time	Cut off	PFS (ETS vs. non‐ETS)	OS (ETS vs. non‐ETS)	mPFS (month) (ETS vs. non‐ETS)	mOS (month) (ETS vs. non‐ETS)	Cut off	PFS (DpR vs. non‐DpR)	OS (DpR vs. non‐DpR)	mPFS (month) (DpR vs. non‐DpR)	mOS (month) (DpR vs. non‐DpR)
G‐SOX [29]	SOX/CS	6 weeks	≥ 20%	HR 0.606 95% CI (0.506–0.725) *p* < 0.0001	HR 0.589 95% CI (0.492–0.704) *p* < 0.0001	4.5 vs. 2.8	14.8 vs. 10.5	As continuous	HR 0.741 95% CI (0.712–0.771) *p* < 0.0001	HR 0.829 95% CI (0.803–0.856) *p* < 0.0001		
Eiji Shinozaki [30]	HER2+: Chemo+trastuzumab	8 weeks	≥ 20%	HR 0.18 *p* < 0.0001	HR 0.25, *p* < 0.0001	11.9 vs. 3.5	24.4 vs. 9.6	44%	HR: 0.22, *p* < 0.0001	HR: 0.24, *p* < 0.0001	14 vs. 5.2	29.7 vs. 11.5
	HER2‐: chemo			HR 0.55, *p* = 0.001	HR 0.69, *p* = 0.059	7.6 vs. 4.0	15.4 vs. 10.4	24%	HR 0.63, *p* = 0.01	HR 0.87 *p* = 0.48	7.6 vs. 4.5	14.8 vs. 12.2

Abbreviations: 95% CI, 95% confidence interval; DpR, depth of response; ETS, early tumor shrinkage; HR, Hazard Ratio; mOS, median overall survival; mPFS, median progression free survival.

### 5.4 Renal Cell Carcinoma

ETS and DpR have been studied in patients using TKIs and antiangiogenic therapy. In the COMPARZ study (NCT00720941), researchers performed a retrospective analysis of 1100 patients to investigate the prognostic value of ETS in patients with renal cell carcinoma receiving first‐line treatment with sunitinib or pazopanib. CT or MRI scans were conducted on days 42 and 90 to assess tumor size changes in SLD. ETS was defined as ≥ 10% reduction. Cox regression analysis showed that ETS ≥ 10% at both days 42 and 90 was associated with better prognosis, and multivariate analysis indicated that ETS ≥ 10% was an independent prognostic factor for OS (Day 42: HR 0.53 95% CI (0.41–0.69), *p* < 0.001, Day 90: HR 0.49 95% CI (0.40, 0.60), *p* < 0.001) [32]. In another study including 4334 patients with metastatic renal cell carcinoma (mRCC), researchers assessed the relationship between ETS and PFS or OS. The study cohort consisted of patients who participated in phase 2 and phase 3 clinical trials between 2003 and 2013, with treatment regimens including axitinib, bevacizumab, interferon alpha, sorafenib, sunitinib, or temsirolimus. The ETS threshold was determined to be 10% through ROC analysis. In the Cox proportional hazards model, patients with ETS showed significantly longer OS (HR 0.615, *p* < 0.0001; median OS: 28.5 vs. 16.0 months) and PFS (HR 0.628, *p* < 0.0001; median PFS: 10.5 vs. 5.3 months) compared to non‐ETS patients [13]. A retrospective analysis further examined 100 mRCC patients who received VEGF‐targeted therapies (including 73 on sunitinib, 12 on sorafenib, 8 on axitinib, 3 on bevacizumab/interferon alpha, and 4 on pazopanib). The study defined EST as the tumor response during the first assessment after two treatment cycles. Patients were classified into different groups based on EST results (−100% to −60%, −59% to −30%, −29% to 0%, 0% to 20%, ≥ 20%). Cox regression analysis showed that EST was an independent prognostic factor for OS (HR 1.624, *p* = 0.021) [33]. In another retrospective study, researchers evaluated the prognosis of 70 mRCC patients receiving first‐line treatment with sunitinib, sorafenib, or bevacizumab. CT data from the first follow‐up (median: 78 days; range: 31–223 days) were collected. ETS was defined as ≥ 10% tumor shrinkage. The results indicated that ETS ≥ 10% could predict longer OS (32.5 months vs. 15.8 months; *p* = 0.002) [34]. However, due to the large follow‐up range (31–223 days) and lack of strict definition for ETS time points, potential biases may affect the reliability and comparability of the results. Furthermore, data obtained from later assessments might lose the early predictive advantage, potentially introducing immortal time bias.

DpR, studies have shown its prognostic value in mRCC patients receiving TKIs or mTOR inhibitors. By analyzing data from 2749 mRCC patients, the role of DpR in predicting patient prognosis was explored. Most patients (84%) had undergone nephrectomy, and 46% had received treatments including sunitinib, sorafenib, axitinib, temsirolimus, etc. Patients were categorized into different groups based on SLD changes (−100% to < −60%, −60% to < −30%, −30% to < 0%, 0% to < +20%, ≥ + 20%). The study also tested whether different tumor shrinkage cutoff points (≥ −10%, ≥ −20%, ≥ −30%) could predict OS and PFS. Survival analysis and Cox regression results indicated that DpR was an independent prognostic factor for OS (HR 0.29, 95% CI (0.22–0.39), *p* < 0.001). In patients with maximum tumor shrinkage (−100% to < −60%), the median OS was 54.5 months, and the median PFS was 17.3 months. As tumor shrinkage decreased, both OS and PFS shortened. When DpR was dichotomized using different cutoff values, patients with DpR showed better prognosis compared to non‐DpR patients. Therefore, DpR can serve as a marker for predicting the efficacy of antitumor treatments [35]. Currently, there are no available studies on the predictive value of ETS and DpR in kidney cancer patients undergoing immunotherapy Table 4.

**TABLE 4:** ETS and DpR in renal cell carcinoma.

Study	Regiment	ETS						DpR				
		ETS time	Cut off	PFS (ETS vs. non‐ETS)	OS (ETS vs. non‐ETS)	mPFS (month) (ETS vs. non‐ETS)	mOS (month) (ETS vs. non‐ETS)	Cut off	PFS (DpR vs. non‐DpR)	OS (DpR vs. non‐DpR)	mPFS (month) (DpR vs. non‐DpR)	mOS (month) (DpR vs. non‐DpR)
COMPARZ [32]	Sunitinib/pazopanib		≥ 10%	—	HR 0.53 95% CI (0.41–0.69), *p* < 0.001							
Viktor Grünwald [13]	Axitinib, bevacizumab, interferon α, sorafenib, sunitinib, temsirolimus		≥ 10%	HR 0.628 95% CI (0.580–0.680), *p* < 0.0001	HR 0.615 *p* < 0.0001	10.5 vs. 5.3	28.5 vs. 16					
C Seidel [33]	Sunitinib sorafenib, axitinib, bevacizumab, interferon α	Two cycles	I (100%–60%), II (59%–30%), III (29%–0%), IV (0%–20%), Gain (> 20%)		HR 1.624 95% CI (1.06–2.09), *p* = 0.021		I: 77.4 II: 33.5 III: 26.9 IV: 29.9 Gain: 14.3					
Toni K. Choueiri [34]	Sunitinib, sorafenib, axitinib, temsirolimus, temsirolimus, interferon‐α.							≥ 30%	HR 1.84 *p* < 0.001	HR 1.50 *p* < 0.001	10.7 vs. 4.5	26.6 vs. 13.7

Abbreviations: 95% CI, 95% confidence interval; DpR, depth of response; ETS, early tumor shrinkage; HR, Hazard Ratio; mOS, median overall survival; mPFS, median progression free survival.

### 5.5 Breast Cancer

The treatment of HER2‐positive metastatic breast cancer (mBC) often involves the use of trastuzumab. In a retrospective study, researchers included 100 HER2‐positive mBC patients who received trastuzumab combined with chemotherapy as first‐line treatment and assessed the predictive role of ETS and DpR on clinical outcomes. The study found that ETS ≥ 30% and DpR ≥ 40% were indicators for PFS, with DpR ≥ 40% also serving as a predictor for OS. Multivariate analysis further suggested that DpR ≥ 40% could act as an independent predictor for both PFS and OS [36].

### 5.6 Cholangiocarcinoma

The JCOG1113 study is a randomized controlled trial for advanced biliary tract cancer, where patients were randomly assigned to receive either GC (gemcitabine + cisplatin) or GS (gemcitabine + S‐1) treatment regimens. The study aimed to evaluate the association between ETS and DpR with clinical outcomes. ETS was defined as a ≥ 20% tumor reduction at week 6, and the cut‐off value for DpR was set at 40%. The results showed that, across all treatment groups, both ETS and DpR were significantly associated with better prognoses. These metrics could serve as effective tools for predicting PFS and OS [37].

### 5.7 Pancreatic Cancer

A retrospective study on pancreatic cancer included 138 patients, 81 of whom received FOLFOXIRI treatment and 57 received GemNab treatment. ETS was defined as a ≥ 20% reduction in SLD between the 6th and 8th week of treatment. The study found that both ETS and DpR were significantly associated with PFS and OS, and were independent prognostic factors. When comparing treatment groups, ETS did not show a difference, but the DpR was significantly better in the three‐drug regimen. Subgroup analysis revealed that in the FOLFOXIRI treatment group, both ETS and DpR were strong predictors of PFS and OS, while in the GemNab group, these two indicators were only associated with OS. Overall, ETS and DpR still have great prognostic potential in first‐line chemotherapy for pancreatic cancer patients [38]. In another retrospective study that included 63 patients with recurrent or metastatic pancreatic cancer, all patients received chemotherapy as first‐line treatment. ETS was defined as a ≥ 20% reduction in the longest diameter of the tumor between the 6th and 12th week. Similar to the first study, both ETS and DpR were associated with better prognosis and were independent predictors of PFS and OS [39]. Similar results have also been validated in other studies related to pancreatic cancer [40].

### 5.8 Esophageal Cancer

A retrospective study on esophageal cancer included 53 patients with metastatic or unresectable esophageal cancer who received pembrolizumab combined with CF (cisplatin and fluorouracil) treatment. The study evaluated the prognostic predictive value of ETS and DpR. ETS was defined as the percentage reduction in the longest diameter of the target lesion compared to baseline at 6 to 8 weeks. The study found that when the cutoff values for ETS and DpR were set at 20% and 30%, respectively, both ETS and DpR were associated with better patient prognosis and were independent predictors of PFS [41]. In the KEYNOTE‐590 study, a subgroup of patients with metastatic esophageal cancer who received pembrolizumab combined with chemotherapy was analyzed. The results indicated that patients with ETS ≥ 20% and DpR ≥ 60% had better PFS and OS [42]. This suggests that ETS and DpR may have predictive value in immunotherapy. Additionally, another study based on the JCOG0807 trial (which investigated the combination of docetaxel, cisplatin, and 5‐fluorouracil (DCF) as first‐line chemotherapy for metastatic esophageal cancer in a phase I/II clinical trial) defined ETS as a ≥ 20% reduction in the sum of the longest diameters of the target lesions after 8 weeks, and DpR as the percentage of the maximum tumor shrinkage during treatment. The study found that patients with ETS had significantly longer PFS and OS. Multivariate analysis indicated that both ETS and DpR were independent factors for prolonged PFS and OS [43]. This result further supports the value of ETS and DpR as survival predictors in esophageal cancer chemotherapy.

### 5.9 Melanoma

In the treatment evaluation of melanoma, the DpR has shown strong prognostic ability for survival. A study that summarized data from BRIM‐2, BRIM‐3, BRIM‐7, and coBRIM trials included patients who all received 960 mg of vemurafenib orally twice daily, with or without 60 mg of cobimetinib orally daily for 21 days, followed by 7 days of treatment interruption. The study results showed that when DpR was treated as a continuous variable, it was significantly associated with PFS and OS, and it was an independent predictor of survival (V group: HR 1.01 95% CI (1.01–1.01), *p* < 0.0001; C + V group: HR 1.02 95% CI (1.01–1.02), *p* < 0.0001). In the combination therapy group, patients exhibited higher levels of DpR. After grouping DpR into quartiles, the patients with the deepest response showed significantly better prognosis, with this difference being more prominent in the dual‐drug treatment group [44]. This result suggests that DpR is an important indicator of treatment response activity, with better response depth significantly improving PFS and OS.

Regarding ETS, researchers analyzed individual patient tumor data from the BRIM‐3 trial (a phase III clinical trial comparing vemurafenib with dacarbazine) as training data and tested the correlation using data from the BRIM‐2 trial (a phase II clinical trial of vemurafenib). ETS was defined as tumor shrinkage within the first 12 weeks of treatment. The researchers attempted to determine a threshold for ETS, but they found that tumor shrinkage from 0% to 100% had a weak correlation with OS and did not predict patient prognosis well [45]. This may be because the ETS assessment in this study was based on the maximum tumor shrinkage at any point during the 12‐week period, rather than at a specific time point as defined in most studies. This approach could have increased patient heterogeneity, making it difficult to identify meaningful thresholds.

### 5.10 Head and Neck Squamous Cell Carcinoma

In a retrospective study, 66 patients with recurrent and metastatic head and neck squamous cell carcinoma (HNSCC) who received pembrolizumab as a first‐line treatment were included. Patients received either pembrolizumab combined with chemotherapy or pembrolizumab monotherapy. DpR was defined as the best tumor change observed at any time point during first‐line treatment (i.e., tumor shrinkage to a negative value). The study results showed that DpR was significantly negatively correlated with survival outcomes (OS: *r* = −0.41, *p* = 0.0017; PFS: *r* = −0.49, *p* < 0.001). In multivariate analysis, patients with DpR ≤ −45% had better OS and PFS, and DpR was identified as an independent predictor of both OS and PFS [46].

### 5.11 Hepatocellular Carcinoma

For patients with hepatocellular carcinoma (HCC) treated with sorafenib, a retrospective analysis based on a multicenter phase II clinical trial (SORAMIC) involving 115 patients showed that ETS (reduction ≥ 20% in tumor diameter at the first follow‐up imaging) was significantly associated with better OS (median 22.1 months vs. 11.4 months, *p* = 0.002) and PFS (median 8.0 months vs. 4.3 months, *p* = 0.034) compared to patients with ETS < 20%. Multivariate analysis further confirmed that ETS was an independent predictor of OS [47]. Another study involving 104 patients with HCC treated with lenvatinib (LEN) also reached similar conclusions, with ETS ≥ 10% significantly correlating with better PFS and OS. Multivariate analysis confirmed that ETS ≥ 10% was an independent predictor of better OS [48].

The prognostic role of ETS in patients with HCC receiving immune therapy has also been validated. A retrospective study included 39 patients receiving immune therapy (either as first‐line or subsequent treatment). The results showed that patients with ETS ≥ 10% had significantly longer survival time after the first follow‐up (15.1 vs. 4.0 months, *p* = 0.008) [49].

### 5.12 Glioma

The predictive value of DpR in recurrent glioblastoma patients receiving anti‐VEGF therapy has been validated. Researchers used data from 276 patients in two phase II clinical trials as a training set (including bevacizumab ± irinotecan (NCT00345163) and cabozantinib (NCT00704288)) and data from 74 patients in another phase III clinical trial as a validation set (bevacizumab [NCT02511405]). T1‐weighted postcontrast images were used to estimate enhanced volume, and a biexponential model was employed to calculate DpR in order to predict survival outcomes for recurrent glioblastoma patients receiving antiangiogenesis therapy. The study results showed that when DpR reached 11.3%, it became a significant predictor of survival (Phase II HR 0.6326, *p* = 0.0028; Phase III HR 0.4785, *p* = 0.0206) [50]. In a retrospective study of the AVAREG phase II randomized trial, the researchers assessed the prognostic role of ETS in patients with recurrent glioblastoma receiving bevacizumab or lomustine. The first disease assessment was conducted on day 46, and MRI images with T1 contrast and T2/FLAIR sequences were used to calculate the relative change in SLD. The optimal ETS cutoff values were determined to be a 15% reduction in T1 contrast and a 40% reduction in T2/FLAIR. The results showed that ETS (15% reduction in T1 contrast) was a significant predictor of OS (HR 0.511, 95% CI (0.269–0.971), *p* = 0.040), whereas no significant correlation was found between T2/FLAIR and OS (*p* = 0.102) [51]. Thus, the ETS evaluation using T1 contrast reduction may serve as a useful survival predictor for recurrent glioblastoma patients treated with bevacizumab. However, this also suggests that the consistency and reliability of different imaging methods used to evaluate ETS need to be considered Table 5.

**TABLE 5:** ETS and DpR in other solid tumors.

Cancer	Study	Patient	Regiment	ETS						DpR				
				ETS Time	Cut off	PFS (ETS vs. non‐ETS)	OS (ETS vs. non‐ETS)	mPFS (month) (ETS vs. non‐ETS)	mOS (month) (ETS vs. non‐ETS)	Cut off	PFS (DpR vs. non‐DpR)	OS (DpR vs. non‐DpR)	mPFS (month) (DpR vs. non‐DpR)	mOS (month) (DpR vs. non‐DpR)
Breast cancer	Luo Y [36]	HER2 positive metastatic breast cancer	Trastuzumab	6 weeks	≥ 30%	HR 0.509 95% CI (0.282–0.916) *p* = 0.024	HR 0.573 95% CI (0.431–2.333) *p* = 0.995	1.43 vs. 0.69 (year)		40%	HR 0.515 95% CI (0.285–0.930) *p* = 0.028	HR 0.367 95% CI (0.149–0.9303) *p* = 0.029	1.43 vs. 0.59 (year)	4.02 vs. 3.07 (year)
Biliary tract cancer	JCOG1113 [37]	Advanced biliary tract cancer	Gemcitabine+S‐1/gemcitabine +cisplatin	6 weeks	≥ 20%	HR 0.70 95% CI (0.52–0.93) *p* = 0.01	HR 0.60 95% CI (0.44–0.81) *p* < 0.01			40%	HR 0.67 95% CI (0.48–0.94) *p* = 0.02	HR 0.64 95% CI (0.46–0.90) *p* < 0.01		
Pancreatic cancer	Caterina Vivaldi [38]	Metastatic pancreatic cancer	FOLFIRINOX/Gemcitabine+Nab‐paclitaxel	6–8 weeks	≥ 20%	HR 1.890 95% CI (1.054–3.389) *p* = 0.033	HR 2.349 95% CI (1.126–4.897) *p* = 0.023	8.0 vs. 4.8	13.2 vs. 9.7	continuous variable	HR 1.035 95% CI (1.022–1.049) *p* < 0.0001	HR 1.038 95% CI (1.022–1.055) *p* < 0.0001	9.0 vs. 6.7	14.3 vs. 11.1
	Meng Qiu [39]	Advanced unresectable pancreatic cancer	Chemochtherapy	6–12 weeks	≥ 20%	HR 4.490 95% CI (1.842–10.946) *p =* 0.001		6.5 vs. 2.2	12.1 vs. 6.0	continuous variable	HR 1.027 95% CI (1.014–1.040) *p* < 0.001	HR 1.037 95% CI (1.024–1.051) *p* < 0.001	9.3 vs. 3.1	18.2 vs. 7.3
	Yu Sunakawa [40]	Advanced pancreatic cancer	FOLFIRINOX	6–8 weeks	≥ 20%	HR 0.37 95% CI (0.16–0.85) *p =* 0.020	HR 0.40 95% CI (0.15–1.06) *p =* 0.065	9.0 vs. 4.2	24.0 vs. 9.1					
Esophageal cancer	Hiroshi Miyata [41]	Metastatic or unresectable locally advanced esophageal cancer	Pembrolizumab plus Chemotherapy	6–8 weeks	≥ 20%	HR 0.15 95% CI (0.06–0.35) *p* ≤ 0.001		12.7 vs. 5.5	14.4 vs. 8.2	30%	HR 0.11 95% CI (0.05–0.26) *p* ≤ 0.001		12.7 vs. 4.9	14.4 vs. 8.0
	KEYNOTE‐590 [42]		Embrolizumab plus chemotherapy	9 weeks	≥ 20%	HR 0.24 95% CI (0.13–0.43)	HR 0.23 95% CI (0.12–0.42)	8.2 vs. 3.9	28.4 vs. 6.4	60%	HR 0.24 95% CI (0.13–0.43)	HR 0.37 95% CI (0.20–0.68)	12.2 vs. 4.3	35.8 vs. 13.9
	JCOG0807 [43]	Metastatic esophageal cancer	Docetaxel combined with cisplatin plus fluorouracil	8 weeks	≥ 20%	HR 0.26 95% CI (0.14–0.49)	HR 0.20 95% CI (0.11–0.39)	7.5 vs. 3.4	13.8 vs. 6.1	30%	HR 0.17 95% CI (0.08–0.34)	HR 0.14 95% CI (0.07–0.27)	7.5 vs. 2.9	13.8 vs. 6.0
Melanoma	Pooled analysis of BRIM‐2, BRIM‐3, BRIM‐7 and coBRIM [44]	BRAFV600‐mutated metastatic melanoma	Vemurafenib or cobimetinib plus vemurafenib							Continuous variable	HR 1.01 95% CI (1.01–1.01) *p* < 0.0001	HR 1.01 95% CI (1.00–1.01) *p* = 0.0003		
											HR 1.02 95% CI (1.01–1.02) *p* < 0.0001	HR 1.01 95% CI (1.00–1.01) *p =* 0.0071		
HCC	SORAMIC [47]	HCC	Sorafenib		≥ 20%		HR 0.44 95% CI (0.24–0.69) *p* < 0.001	8.0 vs. 4.3	22.1 vs. 11.4					
	Michihisa Moriguchi [48]	HCC	Lenvatinib		≥ 10%	HR 0.275 95% CI (0.157–0.483) *p* < 0.001	HR 0.091 95% CI (0.021–0.392) *p =* 0.001	9.3 vs. 3.3	median not reached vs. 12.3					
	Felix Hahn [49]	HCC	Imunotherapy		≥ 10%	HR 26.0 95% CI (2.9–241.0) *p* = 0.004	HR 3.0 95% CI (1.3–6.9) *p* = 0.011	23.6 vs. 2.4	15.1 vs. 4.0					

Abbreviations: 95% CI, 95% confidence interval; DpR, depth of response; ETS, early tumor shrinkage; HR, Hazard Ratio; mOS, median overall survival; mPFS, median progression free survival.

## 6 Heterogeneity and Conflicting Evidence

Across different tumor types and therapeutic contexts, the prognostic and predictive value of ETS and DpR exhibits marked heterogeneity. In metastatic colorectal cancer (mCRC), the TRIBE trial demonstrated that ETS (≥ 20% reduction at 8 weeks) was associated with improved PFS and OS, supporting its role as an early indicator of treatment efficacy and long‐term outcome [9]. In contrast, the FIRE‐3 and related trials showed that patients with RAS wild‐type disease treated with cetuximab achieved higher ETS/DpR rates and longer OS compared with those treated with bevacizumab, highlighting the combined influence of molecular subgroups and therapeutic mechanisms on predictive performance [20, 22]. In EGFR‐mutant NSCLC, analyses from EURTAC and LUX‐Lung 3 failed to demonstrate a consistent association between DpR and long‐term survival [14], whereas immunotherapy trials such as OAK and POPLAR reported that ETS ≥ 10% at week 6 strongly correlated with improved OS and PFS in patients treated with atezolizumab [12]. Furthermore, a retrospective cohort of advanced EGFR‐mutant NSCLC reported a significant association between DpR and PFS [18]. Taken together, these findings indicate that ETS and DpR should be viewed as context‐dependent markers whose predictive utility varies according to tumor biology, drug mechanism, and tumor regression dynamics, rather than universally applicable endpoints.

Several interrelated factors contribute to these discrepancies. Patient‐level heterogeneity—including molecular subtype, disease stage, prior treatment exposure, comorbidities, and baseline tumor burden—modifies therapeutic sensitivity. Treatment modality also shapes tumor regression kinetics: targeted agents often induce rapid but reversible shrinkage, whereas immunotherapies act more slowly, sometimes accompanied by pseudoprogression, altering the mapping of ETS/DpR to survival [12, 14, 20, 22]. Inconsistencies in definitions further compromise comparability: lung cancer studies often applied a 10% ETS threshold [12], whereas mCRC studies used 20% [20, 22], and gastric cancer trials frequently borrowed cutoffs from colorectal cancer despite biological differences [19, 28, 29]. For DpR, some trials defined it as the maximum relative tumor reduction from baseline, while others applied arbitrary quartile groupings [15, 17, 18]. Divergent assessment time points (e.g., 6 vs. 12 weeks) and imaging criteria also risk introducing immortal time bias, artificially inflating associations between early shrinkage and outcomes. Substantial variability in methodological rigor compounds the issue: most evidence derives from retrospective or post hoc analyses of trial datasets, prone to selection and information biases. For example, in mCRC, a large meta‐analysis of 20 randomized controlled trials reported only a weak trial‐level correlation between DpR and OS (R^2^ ≤ 0.21), suggesting that while DpR may be prognostic, it is not a validated surrogate endpoint [27, 28].

Accordingly, ETS and DpR may be best conceptualized as context‐dependent imaging biomarkers: they are clinically useful when applied under homogeneous conditions of treatment mechanism, tumor type, and molecular subgroup with standardized definitions, but their generalizability across heterogeneous settings remains limited. Methodologically, prospective studies should prespecify imaging time points and assessment protocols, model ETS/DpR as continuous variables, and use approaches such as restricted cubic splines or ROC/Youden index to explore data‐driven thresholds with external validation. Landmark or time‐dependent analyses should be employed to mitigate temporal biases, while stratified and interaction analyses by treatment modality (e.g., targeted therapy, immunotherapy, antiangiogenic therapy) and molecular subgroup (e.g., RAS, EGFR) are warranted [9, 12, 14, 20, 22, 28, 29, 30]. Moreover, integrating ETS/DpR with molecular biomarkers, radiomics, and AI‐driven models may enhance robustness and generalizability across centers and platforms. Ultimately, large‐scale, standardized, prospective, multicenter trials are required to validate and calibrate ETS and DpR, thereby defining their appropriate role and boundaries across tumor types and treatment paradigms [27, 28].

## 7 Enhancing the Predictive Power of ETS and DpR

### 7.1 Advancements in Imaging Technology

ETS and DpR in solid tumors are typically evaluated through CT or MRI imaging and interpreted according to the RECIST criteria. Through studies on various tumor types and treatment regimens, we have observed differences in the predictive accuracy of ETS and DpR. These differences may be closely related to multiple factors, including the biological characteristics of the tumor, treatment mechanisms, and the tumor's response to therapy. For example, NSCLC patients with EGFR mutations are highly sensitive to EGFR‐TKIs, with significant tumor shrinkage observed early in treatment [52]. In contrast, immunotherapy activates the host immune system, particularly T cells, to recognize and attack tumor cells, often leading to more sustained immune memory, but its therapeutic response is typically slower and may be accompanied by pseudoprogression [53, 54].

*Fluorodeoxyglucose Positron Emission Tomography Combined with Computed Tomography (FDG PET/CT)* has played an important role in the evaluation of treatment efficacy for various cancers. By assessing the metabolic activity of tumors, FDG PET/CT can provide early indicators of metabolic changes in tumor cells. Since tumor cells typically exhibit higher metabolic activity, and metabolic changes often precede morphological changes during treatment, PET/CT can detect tumor lesions earlier and is more sensitive to treatment responses. Studies have shown that in melanoma patients receiving PD‐1 antibody therapy, changes in the maximum standardized uptake value (SUVmax) on FDG PET/CT scans at 1 week of treatment can predict treatment response. In a cohort of 11 patients, PET/CT scans were performed within 4 weeks before treatment and 1 week after treatment, with CT scans performed every 3 months to assess tumor response according to RECIST criteria. The results showed that 6 patients with a SUVmax increase of over 70% (MF) or a decrease of more than 30% (MR) had a 100% objective response rate (compared to 38% in the non‐MF/MR group), with a PFS of over 38 months (compared to 2.8 months in the non‐MF/MR group). The PFS of the MF–MR group was significantly longer than that of the SM group (*p* = 0.017). Interestingly, during PET/CT detection, the tumor size of MF patients changed very little (range: 0% to 1.3%), while the tumor size of MR patients showed only slight changes (range: −7.7% to 2.9%). This suggests that the sensitivity of PET/CT to metabolic changes may precede conventional CT's assessment of tumor size [55]. Similar studies have been conducted in NSCLC patients receiving nivolumab treatment. A study included 25 advanced NSCLC patients who all received nivolumab treatment. FDG PET/MRI scans were performed at 2 weeks before treatment, 2 weeks after treatment, and 8 weeks after treatment. The researchers extracted total lesion glycolysis (ΔTLG) and apparent diffusion coefficient (ΔADC) values from the SUV changes and MRI scans. They found that patients with a decrease in TLG and an increase in ADC had higher tumor control rates. A combined analysis of ΔTLG and ΔADC revealed that when the cut‐off value was set at 16.5, the median overall survival (OS) of patients with ΔTLG+ΔADC values below 16.5 was significantly longer than that of patients with values ≥ 16.5 (23.6 months vs. 4.7 months, *p* = 0.000154) [56]. These findings suggest that combined PET/CT evaluation of metabolic markers can identify patients who are more likely to respond to treatment at an earlier stage. Although no studies have yet explored the impact of PET/CT on ETS assessment, the unique treatment responses in immunotherapy patients suggest that more comprehensive evaluation metrics should be considered in tumor assessments, especially for those undergoing immunotherapy.

Not All Tumor Treatment Responses Are Reflected Only in Tumor Size. For example, the treatment response in gastrointestinal stromal tumors (GISTs) often manifests early as internal enhancement of the tumor, which may be due to causes like intratumoral bleeding induced by treatment. During the first month, or even within a week of treatment, the CT density of GISTs may change, even when the tumor size does not show significant changes. This poses a challenge to ETS and DpR assessments based on the traditional RECIST criteria. A study on GIST patients treated with imatinib found that 70% of patients with a decrease in SUV on PET/CT compared to baseline showed a ≥ 10% reduction in tumor size or a ≥ 15% decrease in tumor density on CT by the 8th week of treatment [57]. This suggests that PET/CT has high sensitivity in early detection of tumor response in such cases.

#### 7.1.1 MRI

With the deepening of research in tumor pathophysiology, it has become clear that measuring tumor growth and treatment response solely by tumor diameter is inadequate. As a result, more and more studies are focusing on exploring biomarkers that can early reflect the effects of tumor treatments, based on mechanisms of tumor growth, drug functions, and mechanisms of action. For example, treatments targeting tumor angiogenesis, such as VEGFR antibodies and various antiangiogenic TKIs, aim to treat tumors by altering the formation of blood vessels. Imaging techniques like Diffusion Weighted MRI (DWI‐MRI), Dynamic Contrast‐Enhanced MRI (DCE‐MRI), and Dynamic Contrast‐Enhanced CT (DCE‐CT) can sensitively detect changes in blood flow and assist in the early evaluation of tumor treatment response. In patients with recurrent glioma receiving anti‐VEGF treatment, researchers explored whether DWI‐MRI could predict the efficacy of antiangiogenic therapy early. Data from patients in five independent phase II clinical trials who were treated with single‐agent therapy were analyzed. The patients received sunitinib, bevacizumab, cabozantinib, aflibercept, or lomustine. All patients included in the analysis underwent MRI scans, including T1‐weighted and DWI scans, before treatment and within 14 days after treatment initiation. The ADC was calculated. The study found that ADC value was an independent prognostic factor for patient outcomes (HR 0.2734 95% CI (0.1425–0.5244), *p* = 0.0001) [58]. This indicates that MRI can sensitively identify patients who respond to antiangiogenic treatment early. Further studies have also shown that Amide Proton Transfer‐weighted MRI (APT‐MRI) and Perfusion‐weighted MRI (PWI‐MRI) are more sensitive than conventional DWI‐MRI [59]. These findings suggest that not only antiangiogenic therapies but also DWI‐MRI show high sensitivity in early treatment response assessment for cytotoxic chemotherapy [60], radiotherapy [61], anti‐HER2 monoclonal antibody therapy [62] and immunotherapy [63].

#### 7.1.2 Ultrasound

Compared to the high costs of PET/CT and MRI, dynamic contrast‐enhanced ultrasound (DCE‐US) offers a unique advantage as a simple and practical method, especially in providing tumor blood flow and perfusion information. DCE‐US has potential value for early quantitative assessment of treatment response in patients with intrahepatic cholangiocarcinoma (ICC). Researchers classified ICC patients into responders and nonresponders based on the m‐RECIST 1.1 criteria and evaluated the quantitative parameters from DCE‐US scans after 2 months of treatment. The results showed significant reductions in Peak Enhancement (PE), Wash‐in Rate (WiR), Wash‐in Perfusion Index (WiPI), and Wash‐out Rate (WoR) in the responder group (*p* < 0.05). In contrast, in the nonresponder group, only PE and WiPI showed slight decreases (*p* < 0.05) after 2 months of treatment [64]. Another prospective observational study, analyzing patients receiving antiangiogenesis therapy including metastatic breast cancer, metastatic melanoma, metastatic colon cancer, gastrointestinal stromal tumor, metastatic renal cell carcinoma, and primary hepatocellular carcinoma, found that DCE‐US parameters measured on day 7—mean transit time (MTT)—were significantly correlated with PFS (*p* = 0.002), with the most significant results seen in breast cancer (*p* = 0.004) and colon cancer (*p* = 0.025) patients [65]. Additionally, a similar conclusion was drawn in a study on hepatocellular carcinoma, with assessments as early as day 3 posttreatment [66]. The DCE‐US parameters reflect tumor blood vessel normalization, which is one of the mechanisms of antiangiogenic therapy. In certain tumor types, combining these parameters with ETS and DpR may significantly improve the prediction of PFS and OS. However, no studies have yet explored the combined effect of these markers.

#### 7.1.3 Radiomics

In breast cancer patients receiving neoadjuvant therapy, researchers found that different patterns of tumor shrinkage were associated with patient prognosis. Based on MRI imaging analysis, tumors were categorized into concentric shrinkage (CS) and nonconcentric shrinkage (non‐CS) patterns. The study showed that the CS pattern was associated with a better prognosis (median OS of 120.3 months 95% CI (117.8–122.8) vs. 91.5 months 95% CI (85.8–97.2); *p* ≤ 0.001) [67]. This suggests that, in addition to tumor size, the mode, shape, and density of tumor shrinkage should also be comprehensively considered depending on the type of tumor and treatment regimen. Radiomics is a research method that extracts a large number of quantitative features from medical images (such as CT, MRI, PET, etc.) for analysis and data mining. With the development of machine learning and artificial intelligence, radiomics can be combined with genomic, proteomic, and other omics data to provide multidimensional information, thus more accurately predicting patient outcomes and survival. Numerous studies have explored the use of radiomics in predicting efficacy and assessing prognosis in patients receiving immunotherapy. For example, in melanoma patients treated with pembrolizumab, a combination of 25 radiomic features extracted from CT scans predicted OS with an AUC of 0.92 (95% CI, 0.89–0.95), significantly outperforming the AUC predicted by the RECIST standard (0.80, 95% CI, 0.75–0.84) [68]. In advanced nonsmall cell lung cancer patients undergoing chemoradiotherapy, PET/CT radiomics was able to predict PFS. Patients were classified into low‐risk and high‐risk groups based on imaging features, and the 2‐year PFS rates were 61.9% and 33.2%, respectively (*p* = 0.004 log‐rank test; HR, 4.13, 95% CI, 1.42–11.70) [69]. The advantage of radiomics lies in the ability of artificial intelligence and machine learning to not only integrate tumor size features but also analyze multiple imaging features, providing a more comprehensive assessment of patient prognosis and treatment response. However, the manual process of delineating and segmenting tumor lesions is often affected by differences in physician experience and expertise, and it is also time‐consuming. Significant progress in automated lesion detection and recognition could greatly improve the applicability of these techniques and reduce biases.

### 7.2 Use of Biomarkers

In addition to imaging data, the evaluation of ETS and DpR can be combined with blood biomarkers (such as circulating tumor DNA, RNA, etc.), immunohistochemical markers, and molecular imaging data to further improve the prediction accuracy of PFS and OS. Biomarkers help assess the molecular characteristics of tumors and their response to treatment, providing a more comprehensive prognosis evaluation. In immunotherapy research, it has been found that ETS is correlated with the PD‐L1 status of patients when predicting PFS and OS [12]. Therefore, combining other biomarkers with ETS during immunotherapy may enhance its predictive value. In a prospective observational study of patients with unresectable hepatocellular carcinoma treated with lenvatinib, PD‐1 inhibitors, and TACE, the study found that using ETS alone (≥ 10%) could not effectively predict OS. However, when the neutrophil‐to‐lymphocyte ratio (NLR) before treatment was combined with ETS, it better predicted which patients would benefit from treatment. Additionally, patients with lower NLR were more likely to achieve ETS ≥ 10% [70]. In the FIRE‐3 clinical trial, researchers found that the degree of CEA reduction in treated patients was associated with prognosis [71]. CA19‐9 also has predictive value in patients with metastatic pancreatic cancer undergoing chemotherapy [72]. However, in platinum‐resistant ovarian cancer patients, the concordance between CA‐125 and RECIST‐defined PD was poor [73]. Therefore, the selection of these biomarkers should be carefully considered depending on the specific situation.

Circulating tumor DNA (ctDNA) is often used as a reference for assessing treatment response. Simple blood draws can predict the prognosis of cancer patients undergoing anticancer treatment. Compared to imaging examinations, ctDNA can provide prognostic information earlier and more rapidly [74, 75]. The predictive role of ctDNA has been validated in patients with metastatic NSCLC [76, 77], head and neck squamous cell carcinoma (SCCHN), triple‐negative breast cancer (TNBC), high‐grade serous ovarian cancer (HGSOC), malignant melanoma, and mixed solid tumors (MST) [78].

### 7.3 Integrating ETS/DpR With Emerging Biomarkers

Future studies should prioritize integrating ETS and DpR with emerging biomarkers to advance precision response prediction and individualized treatment strategies. Circulating tumor DNA (ctDNA) offers a real‐time reflection of tumor burden, clonal evolution, and resistance mechanisms, often signaling disease progression earlier than imaging modalities [75, 79, 80]. Linking dynamic ctDNA changes with ETS/DpR could substantially enhance the sensitivity and specificity of early treatment assessment [81]. Radiomics, by extracting multidimensional textural and spatial features from CT, MRI, or PET, can reveal tumor heterogeneity and spatial patterns that are beyond the resolution of RECIST, thereby complementing ETS and DpR [82, 83, 84]. Meanwhile, machine learning and artificial intelligence approaches are capable of uncovering complex nonlinear relationships within multimodal datasets, and their integration with ETS/DpR may facilitate the development of more robust and generalizable predictive systems applicable across tumor types and patient populations [85, 86].

Despite these opportunities, several challenges remain. A major barrier is the lack of standardized ctDNA detection platforms and thresholds, which limit reproducibility across studies. Radiomic features are also subject to poor cross‐center repeatability, as differences in imaging scanners, acquisition protocols, and reconstruction algorithms undermine consistency [87]. In the domain of computational modeling, the high dimensionality of imaging features increases the risk of overfitting without robust feature selection or dimensionality reduction methods such as LASSO regression or principal component analysis [86, 88]. Limited sample sizes and scarce external validation further constrain the generalizability and clinical interpretability of current models. Integrating multimodal data—including PET, MRI, CT, clinical, and molecular information—into unified predictive frameworks also remains technically challenging, with few mature platforms available [86, 88]. Additionally, issues of data access and ethics, particularly restrictions on multi‐center data sharing due to privacy regulations and approval processes, continue to impede broader collaboration.

Addressing these challenges will require standardized acquisition and analysis protocols, the establishment of open‐access datasets, multi‐center collaborations, and the development of explainable AI models [86, 87]. A comprehensive, multidimensional, and longitudinal framework that integrates ETS/DpR with ctDNA, radiomics, and AI‐based approaches holds promise for earlier stratification of responders and nonresponders and could accelerate the implementation of precision oncology across diverse tumor contexts [81, 82, 83, 84, 85, 86].

## 8 Conclusions

Tumor efficacy evaluation is a key component in cancer treatment. Compared to OS, PFS can provide earlier insights into the effectiveness of treatment, thus guiding therapeutic decisions. OS has long been regarded as the gold standard for evaluating long‐term patient prognosis. However, as PFS and OS continue to extend, there is an urgent need for markers that can more promptly indicate treatment efficacy and prognosis. Unified and easily accessible indicators can better guide clinical decision‐making. ETS, as an early sensitive marker of tumor treatment, helps to identify patients who are responsive to treatment at an early stage, assisting in treatment decisions and effectively predicting patient survival prognosis. This concept has been confirmed in several retrospective studies. DpR is also significantly correlated with patient survival prognosis, suggesting that reducing tumor burden has a positive impact on long‐term survival and provides a basis for palliative resection in some advanced tumors. The predictive value of both markers has also been confirmed in multiple retrospective studies.

Previous research has shown that differences in tumor types, treatment drugs, and tumor growth dynamics lead to varying performance of ETS and DpR in different situations. Therefore, ETS and DpR provide a measurement dimension for exploring tumor heterogeneity and offer more information for assessing the effects of different treatment drugs. However, ETS and DpR also face many unresolved issues. For example, as continuous variables, ETS and DpR currently lack unified cut‐off values for operational reference, and further exploration is needed. Additionally, it is still unclear whether there exists heterogeneity in non‐ETS and non‐DpR patients. ETS and DpR rely on imaging examination methods to provide accurate measurement parameters. As multidimensional tumor growth evaluation parameters continue to develop, they will help assess patient prognosis more accurately. Advances in imaging technology also provide more precise and multidimensional parameters for measuring ETS and DpR.

In conclusion, as convenient and promising predictive factors, ETS and DpR still require further exploration in larger‐scale prospective clinical studies.

## Author Contributions

**Peng Cao:** writing – original draft preparation. **Xiaojuan Yang:** conceptualization, visualization, supervision, and validation. All authors have read and agreed to the published version of the manuscript.

## Consent

The authors have nothing to report.

## Conflicts of Interest

The authors declare no conflicts of interest.

## Data Availability

Data availability is not applicable to this article as no new data were created or analyzed in this study.

## References

[1] P. A. Tang , S. M. Bentzen , E. X. Chen , and L. L. Siu , “Surrogate End Points for Median Overall Survival in Metastatic Colorectal Cancer: Literature‐Based Analysis From 39 Randomized Controlled Trials of First‐Line Chemotherapy,” Journal of Clinical Oncology 25, no. 29 (2007): 4562–4568. 17876010 10.1200/JCO.2006.08.1935

[2] K. R. Johnson , W. Liauw , and M. N. Lassere , “Evaluating Surrogacy Metrics and Investigating Approval Decisions of Progression‐Free Survival (PFS) in Metastatic Renal Cell Cancer: A Systematic Review,” Annals of Oncology 26, no. 3 (2015): 485–496. 25057168 10.1093/annonc/mdu267

[3] E. A. Eisenhauer , P. Therasse , J. Bogaerts , et al., “New Response Evaluation Criteria in Solid Tumours: Revised RECIST Guideline (Version 1.1),” European Journal of Cancer 45, no. 2 (2009): 228–247. 19097774 10.1016/j.ejca.2008.10.026

[4] P. N. Lara , M. W. Redman , K. Kelly , et al., “Disease Control Rate at 8 Weeks Predicts Clinical Benefit in Advanced Non–Small‐Cell Lung Cancer: Results From Southwest Oncology Group Randomized Trials,” Journal of Clinical Oncology 26, no. 3 (2008): 463–467. 18202421 10.1200/JCO.2007.13.0344

[5] H. J. Prajapati , J. R. Spivey , S. I. Hanish , et al., “mRECIST and EASL Responses at Early Time Point by Contrast‐Enhanced Dynamic MRI Predict Survival in Patients With Unresectable Hepatocellular Carcinoma (HCC) Treated by Doxorubicin Drug‐Eluting Beads Transarterial Chemoembolization (DEB TACE),” Annals of Oncology: Official Journal of the European Society for Medical Oncology 24, no. 4 (2013): 965–973. 23223331 10.1093/annonc/mds605

[6] R. Ricotta , A. Vanzulli , M. Moroni , et al., “Magnetic Resonance Imaging as an Early Indicator of Clinical Outcome in Patients With Metastatic Colorectal Carcinoma Treated With Cetuximab or Panitumumab,” Clinical Colorectal Cancer 12, no. 1 (2013): 45–53. 23041354 10.1016/j.clcc.2012.07.001

[7] C. Suzuki , L. Blomqvist , A. Sundin , et al., “The Initial Change in Tumor Size Predicts Response and Survival in Patients With Metastatic Colorectal Cancer Treated With Combination Chemotherapy,” Annals of Oncology 23, no. 4 (2012): 948–954. 21832285 10.1093/annonc/mdr350

[8] U. Mansmann , U. Sartorius , R. Laubender , C. Giessen , R. Esser , and V. Heinemann , “Quantitative Analysis of the Impact of Deepness of Response on Post‐Progression Survival Time Following First‐Line Treatment in Patients With mCRC,” Annals of Oncology 24 (2013): iv14.

[9] C. Cremolini , F. Loupakis , C. Antoniotti , et al., “Early Tumor Shrinkage and Depth of Response Predict Long‐Term Outcome in Metastatic Colorectal Cancer Patients Treated With First‐Line Chemotherapy Plus Bevacizumab: Results From Phase III TRIBE Trial by the Gruppo Oncologico Del Nord Ovest,” Annals of Oncology: Official Journal of the European Society for Medical Oncology 26, no. 6 (2015): 1188–1194. 25712456 10.1093/annonc/mdv112

[10] G. Fucà , F. Corti , M. Ambrosini , et al., “Prognostic Impact of Early Tumor Shrinkage and Depth of Response in Patients With Microsatellite Instability‐High Metastatic Colorectal Cancer Receiving Immune Checkpoint Inhibitors,” Journal for Immunotherapy of Cancer 9, no. 4 (2021): e002501. 33849927 10.1136/jitc-2021-002501 PMC8051394

[11] H. Piessevaux , M. Buyse , W. De Roock , et al., “Radiological Tumor Size Decrease at Week 6 Is a Potent Predictor of Outcome in Chemorefractory Metastatic Colorectal Cancer Treated With Cetuximab (BOND Trial),” Annals of Oncology: Official Journal of the European Society for Medical Oncology 20, no. 8 (2009): 1375–1382. 19465422 10.1093/annonc/mdp011

[12] A. M. Hopkins , G. Kichenadasse , C. S. Karapetis , A. Rowland , and M. J. Sorich , “Early Tumor Shrinkage Identifies Long‐Term Disease Control and Survival in Patients With Lung Cancer Treated With Atezolizumab,” Journal for Immunotherapy of Cancer 8, no. 1 (2020): e000500. 32503948 10.1136/jitc-2019-000500 PMC7279663

[13] V. Grünwald , X. Lin , D. Kalanovic , and R. Simantov , “Early Tumour Shrinkage: A Tool for the Detection of Early Clinical Activity in Metastatic Renal Cell Carcinoma,” European Urology 70, no. 6 (2016): 1006–1015. 27238653 10.1016/j.eururo.2016.05.010

[14] C. K. Lee , S. Lord , I. Marschner , et al., “The Value of Early Depth of Response in Predicting Long‐Term Outcome in EGFR‐Mutant Lung Cancer,” Journal of Thoracic Oncology 13, no. 6 (2018): 792–800. 29580950 10.1016/j.jtho.2018.03.010

[15] C. E. McCoach , G. M. Blumenthal , L. Zhang , et al., “Exploratory Analysis of the Association of Depth of Response and Survival in Patients With Metastatic Non‐Small‐Cell Lung Cancer Treated With a Targeted Therapy or Immunotherapy,” Annals of Oncology: Official Journal of the European Society for Medical Oncology 28, no. 11 (2017): 2707–2714. 29045514 10.1093/annonc/mdx414 PMC6137816

[16] H. Kawachi , D. Fujimoto , T. Morimoto , et al., “Early Depth of Tumor Shrinkage and Treatment Outcomes in Non‐Small Cell Lung Cancer Treated Using Nivolumab,” Investigational New Drugs 37, no. 6 (2019): 1257–1265. 30937690 10.1007/s10637-019-00770-y

[17] Y. Tachibana , K. Morimoto , T. Yamada , et al., “Depth of Response and Treatment Outcomes of Immune Checkpoint Inhibitor‐Based Therapy in Patients With Advanced Non‐Small Cell Lung Cancer and High PD‐L1 Expression: An Exploratory Analysis of Retrospective Multicenter Cohort,” Investigational New Drugs 42 (2024): 538–546. 39168900 10.1007/s10637-024-01467-7

[18] Y.‐T. Liu , K. Zhang , C.‐C. Li , et al., “Depth of Response Was Associated With Progression‐Free Survival in Patients With Advanced Non‐Small Cell Lung Cancer Treated With EGFR‐TKI,” Journal of Cancer 10, no. 21 (2019): 5108–5113. 31602263 10.7150/jca.33450 PMC6775600

[19] D. Morgensztern , A. Ko , M. O'Brien , et al., “Association Between Depth of Response and Survival in Patients With Advanced‐Stage Non‐Small Cell Lung Cancer Treated With First‐Line Chemotherapy,” Cancer 125, no. 14 (2019): 2394–2399. 30933354 10.1002/cncr.32114

[20] S. Stintzing , D. P. Modest , L. Rossius , et al., “FOLFIRI Plus Cetuximab Versus FOLFIRI Plus Bevacizumab for Metastatic Colorectal Cancer (FIRE‐3): A Post‐Hoc Analysis of Tumour Dynamics in the Final RAS Wild‐Type Subgroup of This Randomised Open‐Label Phase 3 Trial,” Lancet Oncology 17, no. 10 (2016): 1426–1434. 27575024 10.1016/S1470-2045(16)30269-8

[21] H. Piessevaux , M. Buyse , M. Schlichting , et al., “Use of Early Tumor Shrinkage to Predict Long‐Term Outcome in Metastatic Colorectal Cancer Treated With Cetuximab,” Journal of Clinical Oncology 31, no. 30 (2013): 3764–3775. 24043732 10.1200/JCO.2012.42.8532

[22] S. Stintzing , L. Miller‐Phillips , D. P. Modest , et al., “Impact of BRAF and RAS Mutations on First‐Line Efficacy of FOLFIRI Plus Cetuximab Versus FOLFIRI Plus Bevacizumab: Analysis of the FIRE‐3 (AIO KRK‐0306) Study,” European Journal of Cancer 79 (2017): 50–60. 28463756 10.1016/j.ejca.2017.03.023

[23] N. Izawa , K. Shitara , K. Yonesaka , et al., “Early Tumor Shrinkage and Depth of Response in the Second‐Line Treatment for KRAS exon2 Wild‐Type Metastatic Colorectal Cancer: An Exploratory Analysis of the Randomized Phase 2 Trial Comparing Panitumumab and Bevacizumab in Combination With FOLFIRI (WJOG6210G),” Targeted Oncology 15, no. 5 (2020): 623–633. 32960408 10.1007/s11523-020-00750-w

[24] A. Carrato , A. Abad , B. Massuti , et al., “First‐Line Panitumumab Plus FOLFOX4 or FOLFIRI in Colorectal Cancer With Multiple or Unresectable Liver Metastases: A Randomised, Phase II Trial (PLANET‐TTD),” European Journal of Cancer 81 (2017): 191–202. 28633089 10.1016/j.ejca.2017.04.024

[25] P. Manca , S. Corallo , G. Randon , et al., “Impact of Early Tumor Shrinkage and Depth of Response on the Outcomes of Panitumumab‐Based Maintenance in Patients With RAS Wild‐Type Metastatic Colorectal Cancer,” European Journal of Cancer 144 (2021): 31–40. 33321462 10.1016/j.ejca.2020.11.017

[26] G. Sommerhäuser , A. Kurreck , A. Beck , et al., “Depth of Response of Induction Therapy and Consecutive Maintenance Treatment in Patients With RAS Wild‐Type Metastatic Colorectal Cancer: An Analysis of the Panama Trial (AIO KRK 0212),” European Journal of Cancer 178 (2023): 37–48. 36399909 10.1016/j.ejca.2022.09.011

[27] J. D. Wolchok , A. Hoos , S. O'Day , et al., “Guidelines for the Evaluation of Immune Therapy Activity in Solid Tumors: Immune‐Related Response Criteria,” Clinical Cancer Research 15, no. 23 (2009): 7412–7420. 19934295 10.1158/1078-0432.CCR-09-1624

[28] T. Burzykowski , E. Coart , E. D. Saad , et al., “Evaluation of Continuous Tumor‐Size‐Based End Points as Surrogates for Overall Survival in Randomized Clinical Trials in Metastatic Colorectal Cancer,” JAMA Network Open 2, no. 9 (2019): e1911750. 31539075 10.1001/jamanetworkopen.2019.11750 PMC6755539

[29] T. Nishina , M. Azuma , K. Nishikawa , et al., “Early Tumor Shrinkage and Depth of Response in Patients With Advanced Gastric Cancer: A Retrospective Analysis of a Randomized Phase III Study of First‐Line S‐1 Plus Oxaliplatin vs. S‐1 Plus Cisplatin,” Gastric Cancer 22, no. 1 (2019): 138–146. 29948386 10.1007/s10120-018-0845-7

[30] H. Osumi , D. Takahari , E. Shinozaki , et al., “Associations Between Early Tumor Shrinkage and Depth of Response and Clinical Outcomes in Patients Treated With 1st‐Line Chemotherapy for Advanced Gastric Cancer,” Gastric Cancer 21, no. 2 (2018): 267–275. 28584889 10.1007/s10120-017-0729-2

[31] N. Zhu , J. Q. Chen , M. Y. Yang , Y. Cheng , and Y. Yuan , “Relationship of Early Tumor Shrinkage and Depth of Response With the Prognosis and Treatment Effect of Trastuzumab Combined With Chemotherapy as First‐Line Treatment in Advanced Gastric Cancer Patients With Epidermal Growth Factor Receptor 2 Positive,” Zhonghua Zhong Liu Za Zhi 42, no. 10 (2020): 869–875. 33113630 10.3760/cma.j.cn112152-20190213-00082

[32] V. Grünwald , M. Dietrich , and G. R. Pond , “Early Tumor Shrinkage Is Independently Associated With Improved Overall Survival Among Patients With Metastatic Renal Cell Carcinoma: A Validation Study Using the COMPARZ Cohort,” World Journal of Urology 36, no. 9 (2018): 1423–1429. 29654533 10.1007/s00345-018-2297-4

[33] C. Seidel , J. Busch , S. Weikert , S. Steffens , C. Bokemeyer , and V. Grünwald , “Tumour Shrinkage Measured With First Treatment Evaluation Under VEGF‐Targeted Therapy as Prognostic Marker in Metastatic Renal Cell Carcinoma (mRCC),” British Journal of Cancer 109, no. 12 (2013): 2998–3004. 24169357 10.1038/bjc.2013.662 PMC3859945

[34] K. M. Krajewski , M. Guo , A. D. Van den Abbeele , et al., “Comparison of Four Early Posttherapy Imaging Changes (EPTIC; RECIST 1.0, Tumor Shrinkage, Computed Tomography Tumor Density, Choi Criteria) in Assessing Outcome to Vascular Endothelial Growth Factor‐Targeted Therapy in Patients With Advanced Renal Cell Carcinoma,” European Urology 59, no. 5 (2011): 856–862. 21306819 10.1016/j.eururo.2011.01.038

[35] V. Grünwald , R. R. McKay , K. M. Krajewski , et al., “Depth of Remission Is a Prognostic Factor for Survival in Patients With Metastatic Renal Cell Carcinoma,” European Urology 67, no. 5 (2015): 952–958. 25577718 10.1016/j.eururo.2014.12.036 PMC4570832

[36] Y.‐Q. Che , Y. Zhang , K.‐P. Ou , et al., “Depth of Response and Early Tumor Shrinkage for Predicting Clinical Outcomes in HER2‐Positive Metastatic Breast Cancer Treated With Trastuzumab,” Cancer Management and Research 12 (2020): 8527–8534. 32982445 10.2147/CMAR.S269067 PMC7502405

[37] N. Okano , C. Morizane , T. Okusaka , et al., “Early Tumor Shrinkage and Depth of Response as Predictors of Survival for Advanced Biliary Tract Cancer: An Exploratory Analysis of JCOG1113,” Oncologist 29, no. 1 (2024): e97–e107. 37531645 10.1093/oncolo/oyad220 PMC10769805

[38] C. Vivaldi , L. Fornaro , C. Cappelli , et al., “Early Tumor Shrinkage and Depth of Response Evaluation in Metastatic Pancreatic Cancer Treated With First Line Chemotherapy: An Observational Retrospective Cohort Study,” Cancers (Basel) 11, no. 7 (2019): 939. 31277449 10.3390/cancers11070939 PMC6678367

[39] X. Yang , X. Xian , Y. Wang , and M. Qiu , “Assessing Prognostic Value of Early Tumor Shrinkage and Depth of Response in First‐Line Therapy for Patients With Advanced Unresectable Pancreatic Cancer,” BMC Gastroenterology 21, no. 1 (2021): 294. 34266410 10.1186/s12876-021-01870-x PMC8281486

[40] Y. Kaga , Y. Sunakawa , Y. Kubota , et al., “Early Tumor Shrinkage as a Predictor of Favorable Outcomes in Patients With Advanced Pancreatic Cancer Treated With FOLFIRINOX,” Oncotarget 7, no. 41 (2016): 67314–67320. 27634903 10.18632/oncotarget.12007 PMC5341877

[41] T. Sugase , T. Kanemura , T. Takeoka , et al., “Clinical Impact of Early Tumour Shrinkage in Metastatic or Unresectable Oesophageal Cancer Treated With Pembrolizumab Plus Chemotherapy,” Oncology 102, no. 6 (2024): 484–493. 38052183 10.1159/000535186 PMC11152033

[42] K. Kato , T. Kojima , H. Hara , et al., “First‐Line Pembrolizumab Plus Chemotherapy for Advanced/Metastatic Esophageal Cancer: 1‐Year Extended Follow‐Up in the Japanese Subgroup of the Phase 3 KEYNOTE‐590 Study,” Esophagus 21, no. 3 (2024): 306–318. 38607538 10.1007/s10388-024-01053-z PMC11199245

[43] T. Ura , S. Hironaka , Y. Tsubosa , et al., “Early Tumor Shrinkage and Depth of Response in Patients With Metastatic Esophageal Cancer Treated With 2‐Weekly Docetaxel Combined With Cisplatin Plus Fluorouracil: An Exploratory Analysis of the JCOG0807,” Esophagus 20, no. 2 (2023): 272–280. 36427158 10.1007/s10388-022-00968-9

[44] K. D. Lewis , J. Larkin , A. Ribas , et al., “Impact of Depth of Response on Survival in Patients Treated With Cobimetinib ± Vemurafenib: Pooled Analysis of BRIM‐2, BRIM‐3, BRIM‐7 and coBRIM,” British Journal of Cancer 121, no. 7 (2019): 522–528. 31417188 10.1038/s41416-019-0546-y PMC6889491

[45] E. C. Zabor , G. Heller , L. H. Schwartz , and P. B. Chapman , “Correlating Surrogate Endpoints With Overall Survival at the Individual Patient Level in BRAFV600E‐Mutated Metastatic Melanoma Patients Treated With Vemurafenib,” Clinical Cancer Research 22, no. 6 (2016): 1341–1347. 26490313 10.1158/1078-0432.CCR-15-1441 PMC5500836

[46] K. Saijo , H. Imai , K. Ouchi , et al., “Depth of Response May Predict Clinical Outcome in Patients With Recurrent/Metastatic Head and Neck Cancer Treated With Pembrolizumab‐Containing Regimens,” Frontiers in Oncology 13 (2023): 1230731. 37664016 10.3389/fonc.2023.1230731 PMC10469278

[47] O. Öcal , R. Schinner , K. Schütte , et al., “Early Tumor Shrinkage and Response Assessment According to mRECIST Predict Overall Survival in Hepatocellular Carcinoma Patients Under Sorafenib,” Cancer Imaging 22, no. 1 (2022): 1. 34983668 10.1186/s40644-021-00439-x PMC8725442

[48] A. Takahashi , M. Moriguchi , Y. Seko , et al., “Early Tumor Shrinkage as a Predictive Factor for Outcomes in Hepatocellular Carcinoma Patients Treated With Lenvatinib: A Multicenter Analysis,” Cancers 12, no. 3 (2020): 754. 32209994 10.3390/cancers12030754 PMC7140019

[49] L. Müller , S. J. Gairing , R. Kloeckner , et al., “The Prognostic Role of Early Tumor Shrinkage in Patients With Hepatocellular Carcinoma Undergoing Immunotherapy,” Cancer Imaging 22, no. 1 (2022): 54. 36153569 10.1186/s40644-022-00487-x PMC9509639

[50] B. M. Ellingson , A. Hagiwara , C. J. Morris , et al., “Depth of Radiographic Response and Time to Tumor Regrowth Predicts Overall Survival Following Anti‐VEGF Therapy in Recurrent Glioblastoma,” Clinical Cancer Research: An Official Journal of the American Association for Cancer Research 29, no. 20 (2023): 4186–4195. 37540556 10.1158/1078-0432.CCR-23-1235 PMC10592195

[51] A. A. Brandes , G. Finocchiaro , V. Zagonel , et al., “Early Tumour Shrinkage as a Survival Predictor in Patients With Recurrent Glioblastoma Treated With Bevacizumab in the AVAREG Randomized Phase II Study,” Oncotarget 8, no. 33 (2017): 55575–55581. 28903444 10.18632/oncotarget.15735 PMC5589683

[52] G. da Cunha Santos , F. A. Shepherd , and M. S. Tsao , “EGFR Mutations and Lung Cancer,” Annual Review of Pathology 6 (2011): 49–69. 10.1146/annurev-pathol-011110-130206 20887192

[53] H. J. Park , K. W. Kim , J. Pyo , et al., “Incidence of Pseudoprogression During Immune Checkpoint Inhibitor Therapy for Solid Tumors: A Systematic Review and Meta‐Analysis,” Radiology 297, no. 1 (2020): 87–96. 32749204 10.1148/radiol.2020200443 PMC7526949

[54] E. S. Tsang and L. L. Siu , “Peeling the I‐Onion to Demystify Pseudoprogression,” Cancer Cell 41, no. 9 (2023): 1545–1547. 37699332 10.1016/j.ccell.2023.08.003

[55] T. M. Anderson , B. H. Chang , A. C. Huang , et al., “FDG PET/CT Imaging 1 Week After a Single Dose of Pembrolizumab Predicts Treatment Response in Patients With Advanced Melanoma,” Clinical Cancer Research: An Official Journal of the American Association for Cancer Research 30, no. 9 (2024): 1758–1767. 38263597 10.1158/1078-0432.CCR-23-2390 PMC11062839

[56] Y. Umeda , M. Morikawa , M. Anzai , et al., “Predictive Value of Integrated 18F‐FDG PET/MRI in the Early Response to Nivolumab in Patients With Previously Treated Non‐Small Cell Lung Cancer,” Journal for Immunotherapy of Cancer 8, no. 1 (2020): e000349. 32345624 10.1136/jitc-2019-000349 PMC7213911

[57] H. Choi , C. Charnsangavej , S. C. Faria , et al., “Correlation of Computed Tomography and Positron Emission Tomography in Patients With Metastatic Gastrointestinal Stromal Tumor Treated at a Single Institution With Imatinib Mesylate: Proposal of New Computed Tomography Response Criteria,” Journal of Clinical Oncology 25, no. 13 (2007): 1753–1759. 17470865 10.1200/JCO.2006.07.3049

[58] B. M. Ellingson , E. R. Gerstner , M. Smits , et al., “Diffusion MRI Phenotypes Predict Overall Survival Benefit From Anti‐VEGF Monotherapy in Recurrent Glioblastoma: Converging Evidence From Phase II Trials,” Clinical Cancer Research 23, no. 19 (2017): 5745–5756. 28655794 10.1158/1078-0432.CCR-16-2844 PMC5626594

[59] J. E. Park , H. S. Kim , S. Y. Park , S. C. Jung , J. H. Kim , and H.‐Y. Heo , “Identification of Early Response to Anti‐Angiogenic Therapy in Recurrent Glioblastoma: Amide Proton Transfer‐Weighted and Perfusion‐Weighted MRI Compared With Diffusion‐Weighted MRI,” Radiology 295, no. 2 (2020): 397–406. 32154775 10.1148/radiol.2020191376

[60] J. H. Kim , I. Joo , T.‐Y. Kim , et al., “Diffusion‐Related MRI Parameters for Assessing Early Treatment Response of Liver Metastases to Cytotoxic Therapy in Colorectal Cancer,” AJR. American Journal of Roentgenology 207, no. 3 (2016): W26–W32. 27303858 10.2214/AJR.15.15683

[61] X. Li , F. Yuan , L. Ni , and X. Li , “Meta‐Analysis of MRI in Predicting Early Response to Radiotherapy and Chemotherapy in Esophageal Cancer,” Academic Radiology 32 (2024): 798–812. 39266443 10.1016/j.acra.2024.08.055

[62] A. van der Voort , K. J. J. van der Hoogt , R. Wessels , et al., “Diffusion‐Weighted Imaging in Addition to Contrast‐Enhanced MRI in Identifying Complete Response in HER2‐Positive Breast Cancer,” European Radiology 34, no. 12 (2024): 7994–8004. 38967659 10.1007/s00330-024-10857-7 PMC11557627

[63] W. Li , N. N. Le , N. Onishi , et al., “Diffusion‐Weighted MRI for Predicting Pathologic Complete Response in Neoadjuvant Immunotherapy,” Cancers (Basel) 14, no. 18 (2022): 4436. 36139594 10.3390/cancers14184436 PMC9497087

[64] X.‐Y. Lu , J. Jiang , S. Chen , et al., “Application of Dynamic Contrast Enhanced Ultrasound Analysis in Predicting Early Response to Systemic Therapy of Intrahepatic Cholangiocarcinoma,” European Journal of Radiology 175 (2024): 111439. 38547743 10.1016/j.ejrad.2024.111439

[65] N. Lassau , B. Coiffier , M. Kind , et al., “Selection of an Early Biomarker for Vascular Normalization Using Dynamic Contrast‐Enhanced Ultrasonography to Predict Outcomes of Metastatic Patients Treated With Bevacizumab,” Annals of Oncology: Official Journal of the European Society for Medical Oncology 27, no. 10 (2016): 1922–1928. 27502701 10.1093/annonc/mdw280 PMC5035788

[66] N. Lassau , S. Koscielny , L. Chami , et al., “Advanced Hepatocellular Carcinoma: Early Evaluation of Response to Bevacizumab Therapy at Dynamic Contrast‐Enhanced US With Quantification—Preliminary Results,” Radiology 258, no. 1 (2011): 291–300. 20980447 10.1148/radiol.10091870

[67] I. Fukada , K. Araki , K. Kobayashi , et al., “Pattern of Tumor Shrinkage During Neoadjuvant Chemotherapy Is Associated With Prognosis in Low‐Grade Luminal Early Breast Cancer,” Radiology 286, no. 1 (2018): 49–57. 28737968 10.1148/radiol.2017161548

[68] L. Dercle , B. Zhao , M. Gönen , et al., “Early Readout on Overall Survival of Patients With Melanoma Treated With Immunotherapy Using a Novel Imaging Analysis,” JAMA Oncology 8, no. 3 (2022): 385–392. 35050320 10.1001/jamaoncol.2021.6818 PMC8778619

[69] N. Zhang , R. Liang , M. F. Gensheimer , et al., “Early Response Evaluation Using Primary Tumor and Nodal Imaging Features to Predict Progression‐Free Survival of Locally Advanced Non‐Small Cell Lung Cancer,” Theranostics 10, no. 25 (2020): 11707–11718. 33052242 10.7150/thno.50565 PMC7546006

[70] S. Qu , D. Wu , and Z. Hu , “Neutrophil‐To‐Lymphocyte Ratio and Early Tumor Shrinkage as Predictive Biomarkers in Unresectable Hepatocellular Carcinoma Patients Treated With Lenvatinib, PD‐1 Inhibitors, in Combination With TACE,” Technology in Cancer Research & Treatment 22 (2023): 15330338231206704. 37849287 10.1177/15330338231206704 PMC10585992

[71] M. Michl , S. Stintzing , L. Fischer von Weikersthal , et al., “CEA Response Is Associated With Tumor Response and Survival in Patients With KRAS Exon 2 Wild‐Type and Extended RAS Wild‐Type Metastatic Colorectal Cancer Receiving First‐Line FOLFIRI Plus Cetuximab or Bevacizumab (FIRE‐3 Trial),” Annals of Oncology: Official Journal of the European Society for Medical Oncology 27, no. 8 (2016): 1565–1572. 27234640 10.1093/annonc/mdw222

[72] E. G. Chiorean , D. D. Von Hoff , M. Reni , et al., “CA19‐9 Decrease at 8 Weeks as a Predictor of Overall Survival in a Randomized Phase III Trial (MPACT) of Weekly Nab‐Paclitaxel Plus Gemcitabine Versus Gemcitabine Alone in Patients With Metastatic Pancreatic Cancer,” Annals of Oncology: Official Journal of the European Society for Medical Oncology 27, no. 4 (2016): 654–660. 26802160 10.1093/annonc/mdw006 PMC4803454

[73] K. Lindemann , G. Kristensen , M. R. Mirza , et al., “Poor Concordance Between CA‐125 and RECIST at the Time of Disease Progression in Patients With Platinum‐Resistant Ovarian Cancer: Analysis of the AURELIA Trial,” Annals of Oncology: Official Journal of the European Society for Medical Oncology 27, no. 8 (2016): 1505–1510. 27407100 10.1093/annonc/mdw238

[74] R. B. Corcoran and B. A. Chabner , “Application of Cell‐Free DNA Analysis to Cancer Treatment,” New England Journal of Medicine 379, no. 18 (2018): 1754–1765. 30380390 10.1056/NEJMra1706174

[75] J. C. M. Wan , C. Massie , J. Garcia‐Corbacho , et al., “Liquid Biopsies Come of Age: Towards Implementation of Circulating Tumour DNA,” Nature Reviews. Cancer 17, no. 4 (2017): 223–238. 28233803 10.1038/nrc.2017.7

[76] Z. J. F. Assaf , W. Zou , A. D. Fine , et al., “A Longitudinal Circulating Tumor DNA‐Based Model Associated With Survival in Metastatic Non‐Small‐Cell Lung Cancer,” Nature Medicine 29, no. 4 (2023): 859–868. 10.1038/s41591-023-02226-6 PMC10115641 36928816

[77] B. Ricciuti , G. Jones , M. Severgnini , et al., “Early Plasma Circulating Tumor DNA (ctDNA) Changes Predict Response to First‐Line Pembrolizumab‐Based Therapy in Non‐Small Cell Lung Cancer (NSCLC),” Journal for Immunotherapy of Cancer 9, no. 3 (2021): e001504. 33771889 10.1136/jitc-2020-001504 PMC7996662

[78] S. V. Bratman , S. Y. C. Yang , M. A. J. Iafolla , et al., “Personalized Circulating Tumor DNA Analysis as a Predictive Biomarker in Solid Tumor Patients Treated With Pembrolizumab,” Nature Cancer 1, no. 9 (2020): 873–881. 35121950 10.1038/s43018-020-0096-5

[79] C. Abbosh , N. J. Birkbak , G. A. Wilson , et al., “Phylogenetic ctDNA Analysis Depicts Early‐Stage Lung Cancer Evolution,” Nature 545, no. 7655 (2017): 446–451. 28445469 10.1038/nature22364 PMC5812436

[80] J. D. Cohen , L. Li , Y. Wang , et al., “Detection and Localization of Surgically Resectable Cancers With a Multi‐Analyte Blood Test,” Science 359, no. 6378 (2018): 926–930. 29348365 10.1126/science.aar3247 PMC6080308

[81] Y. K. Chae and M. S. Oh , “Detection of Minimal Residual Disease Using ctDNA in Lung Cancer: Current Evidence and Future Directions,” Journal of Thoracic Oncology 14, no. 1 (2019): 16–24. 30296486 10.1016/j.jtho.2018.09.022

[82] P. Lambin , R. T. H. Leijenaar , T. M. Deist , et al., “Radiomics: The Bridge Between Medical Imaging and Personalized Medicine,” Nature Reviews Clinical Oncology 14, no. 12 (2017): 749–762. 10.1038/nrclinonc.2017.141 28975929

[83] H. J. Aerts , E. R. Velazquez , R. T. Leijenaar , et al., “Decoding Tumour Phenotype by Noninvasive Imaging Using a Quantitative Radiomics Approach,” Nature Communications 5 (2014): 4006. 10.1038/ncomms5006 PMC4059926 24892406

[84] R. Sun , E. J. Limkin , M. Vakalopoulou , et al., “A Radiomics Approach to Assess Tumour‐Infiltrating CD8 Cells and Response to Anti‐PD‐1 or Anti‐PD‐L1 Immunotherapy: An Imaging Biomarker, Retrospective Multicohort Study,” Lancet Oncology 19, no. 9 (2018): 1180–1191. 30120041 10.1016/S1470-2045(18)30413-3

[85] A. Esteva , B. Kuprel , R. A. Novoa , et al., “Dermatologist‐Level Classification of Skin Cancer With Deep Neural Networks,” Nature 542, no. 7639 (2017): 115–118. 28117445 10.1038/nature21056 PMC8382232

[86] A. Hosny , C. Parmar , J. Quackenbush , L. H. Schwartz , and H. Aerts , “Artificial Intelligence in Radiology,” Nature Reviews Cancer 18, no. 8 (2018): 500–510. 29777175 10.1038/s41568-018-0016-5 PMC6268174

[87] A. Zwanenburg , M. Vallières , M. A. Abdalah , et al., “The Image Biomarker Standardization Initiative: Standardized Quantitative Radiomics for High‐Throughput Image‐Based Phenotyping,” Radiology 295, no. 2 (2020): 328–338. 32154773 10.1148/radiol.2020191145 PMC7193906

[88] S. S. Yip and H. J. Aerts , “Applications and Limitations of Radiomics,” Physics in Medicine and Biology 61, no. 13 (2016): R150–R166. 27269645 10.1088/0031-9155/61/13/R150 PMC4927328

